# Causal effects of systemic inflammatory proteins on Guillain-Barre Syndrome: insights from genome-wide Mendelian randomization, single-cell RNA sequencing analysis, and network pharmacology

**DOI:** 10.3389/fimmu.2024.1456663

**Published:** 2024-09-09

**Authors:** Jingwen Liu, Renbing Pan

**Affiliations:** ^1^ Department of Neurology and Psychiatry, Longyou People’s Hospital Affiliated with Sir Run Run Shaw Hospital, Zhejiang University School of Medicine, Quzhou, Zhejiang, China; ^2^ Department of Urology, The Quzhou Affiliated Hospital of Wenzhou Medical University, Quzhou People’s Hospital, Quzhou, Zhejiang, China

**Keywords:** systemic inflammatory proteins, Guillain-Barre Syndrome, Mendelian randomization, single-cell RNA-seq, molecular docking

## Abstract

**Background:**

Evidence from observational studies indicates that inflammatory proteins play a vital role in Guillain-Barre Syndrome (GBS). Nevertheless, it is unclear how circulating inflammatory proteins are causally associated with GBS. Herein, we conducted a two-sample Mendelian randomization (MR) analysis to systematically explore the causal links of genetically determined systemic inflammatory proteins on GBS.

**Methods:**

A total of 8,293 participants of European ancestry were included in a genome-wide association study of 41 inflammatory proteins as instrumental variables. Five MR approaches, encompassing inverse-variance weighted, weighted median, MR-Egger, simple model, and weighted model were employed to explore the causal links between inflammatory proteins and GBS. MR-Egger regression was utilized to explore the pleiotropy. Cochran’s Q statistic was implemented to quantify the heterogeneity. Furthermore, we performed single-cell RNA sequencing analysis and predicted potential drug targets through molecular docking technology.

**Results:**

By applying MR analysis, four inflammatory proteins causally associated with GBS were identified, encompassing IFN-γ (OR:1.96, 95%CI: 1.02-3.78, P_IVW_=0.045), IL-7 (OR:1.86, 95%CI: 1.07-3.23, P_IVW_=0.029), SCGF-β (OR:1.56, 95%CI: 1.11-2.19, P_IVW_=0.011), and Eotaxin (OR:1.99, 95%CI: 1.01-3.90, P_IVW_=0.046). The sensitivity analysis revealed no evidence of pleiotropy or heterogeneity. Additionally, significant genes were found through single-cell RNA sequencing analysis and several anti-inflammatory or neuroprotective small molecular compounds were identified by utilizing molecular docking technology.

**Conclusions:**

Our MR analysis suggested that IFN-γ, IL-7, SCGF-β, and Eotaxin were causally linked to the occurrence and development of GBS. These findings elucidated potential causal associations and highlighted the significance of these inflammatory proteins in the pathogenesis and prospective therapeutic targets for GBS.

## 1 Introduction

Guillain-Barre Syndrome (GBS), also known as acute inflammatory demyelinating polyneuropathy, is an autoimmune disorder affecting the peripheral nervous system. It is primarily triggered by an abnormal immune response to an infectious pathogen (1). The clinical manifestations encompass a progressive, symmetrical paralysis that ascends, accompanied by flaccid limbs and varying degrees of sensory impairments (2). The pathogenesis of GBS remains largely elusive, however, it has been established that the immune inflammatory response plays a crucial role in the onset and progression of this condition (3).

Currently, accumulating evidence suggests that systemic and local release of inflammatory cytokines as well as their involvement in immune-mediated demyelination and axonal damage of peripheral nerves, play a crucial role in the pathogenesis of GBS (4). A previous study has demonstrated that inflammatory proteins play a vital role in the occurrence and biological mechanism of GBS and its experimental autoimmune neuritis in animal models (5). Another previous study has demonstrated that, in GBS, inflammatory cytokines can attract effector cells to the peripheral nerve roots, thereby triggering the production of nitric oxide, oxygen free radicals, and other substances that contribute to the demyelination of nerve roots (6). Interestingly, experimental research has manifested that serum circulating levels of IL-6 and TNF-α are higher than normal in individuals with GBS, thereby predicting the disease severity and other clinical characteristics of GBS (3). In addition, another research conducted by A. R. Exley et al. revealed that raised TNF-α concentrations were found in the GBS patients, suggesting that TNF-α may be a vital factor in the pathogenesis of GBS (7). In contrast, another study performed by E. Doncel-Perez et al. suggested that the levels of IL-2, TNF- β, and TNF-α were significantly lower in GBS individuals when compared with control participants, indicating an association between early growth response gene-2 (EGR2) and these inflammatory regulators in the recovery of GBS (8). Nevertheless, whether these inflammatory proteins have causal associations with GBS remains to be elucidated. Thus, given the susceptibility of observational studies to underlying control bias and confounders (9), further investigation into the potential causal effects of genetically predicted circulating inflammatory proteins and GBS is still required.

Mendelian randomization (MR) analysis can provide an effective means to assess causal inferences in observational studies (10). Conducting randomized controlled trials (RCTs) to detect the influence of circulating inflammatory proteins on the risk of GBS is impractical resulting from ethical conditions. Furthermore, the causal links of circulating inflammatory proteins on GBS remain vague in observational research on account of potential confounding factors. Therefore, alternative methods that facilitate casual inference can offer prospective insights into whether particular inflammatory proteins represent underlying risky factors. Conceptually, MR shares similarities with RCTs as genetic variants are randomly assigned before birth, thereby minimizing confounders and reducing reverse causality bias (11). For example, a previous MR research demonstrated that genetically determined levels of specific inflammatory cytokines exhibited causal associations with the risk of ischemic stroke (12). Furthermore, another MR study revealed that targeted interventions of relevant inflammatory regulators could reduce the risk of proliferative diabetic retinopathy (13).

As of now, existing research evidence is restricted to the effect of a single inflammatory protein on GBS outcome, whether from observational studies or RCTs. To date, the causal links between circulating inflammatory proteins and GBS have yet to be established through MR study. Hence, in the present study, by performing a two-sample MR and single-cell RNA sequencing analysis, we would methodically estimate the potential causal associations between genetically determined circulating inflammatory proteins and GBS. By integrating the findings of observational studies, MR analyses, and single-cell RNA sequencing analysis, we are expected to elucidate the underlying causal roles of specific circulating inflammatory proteins in the pathogenesis of GBS. Moreover, understanding the intricate correlations between systemic inflammatory proteins and the occurrence and progression in depth may be attributed to the development of targeted therapeutic interventions for GBS in the future.

## 2 Materials and methods

### 2.1 Study design

**Figure 1:**
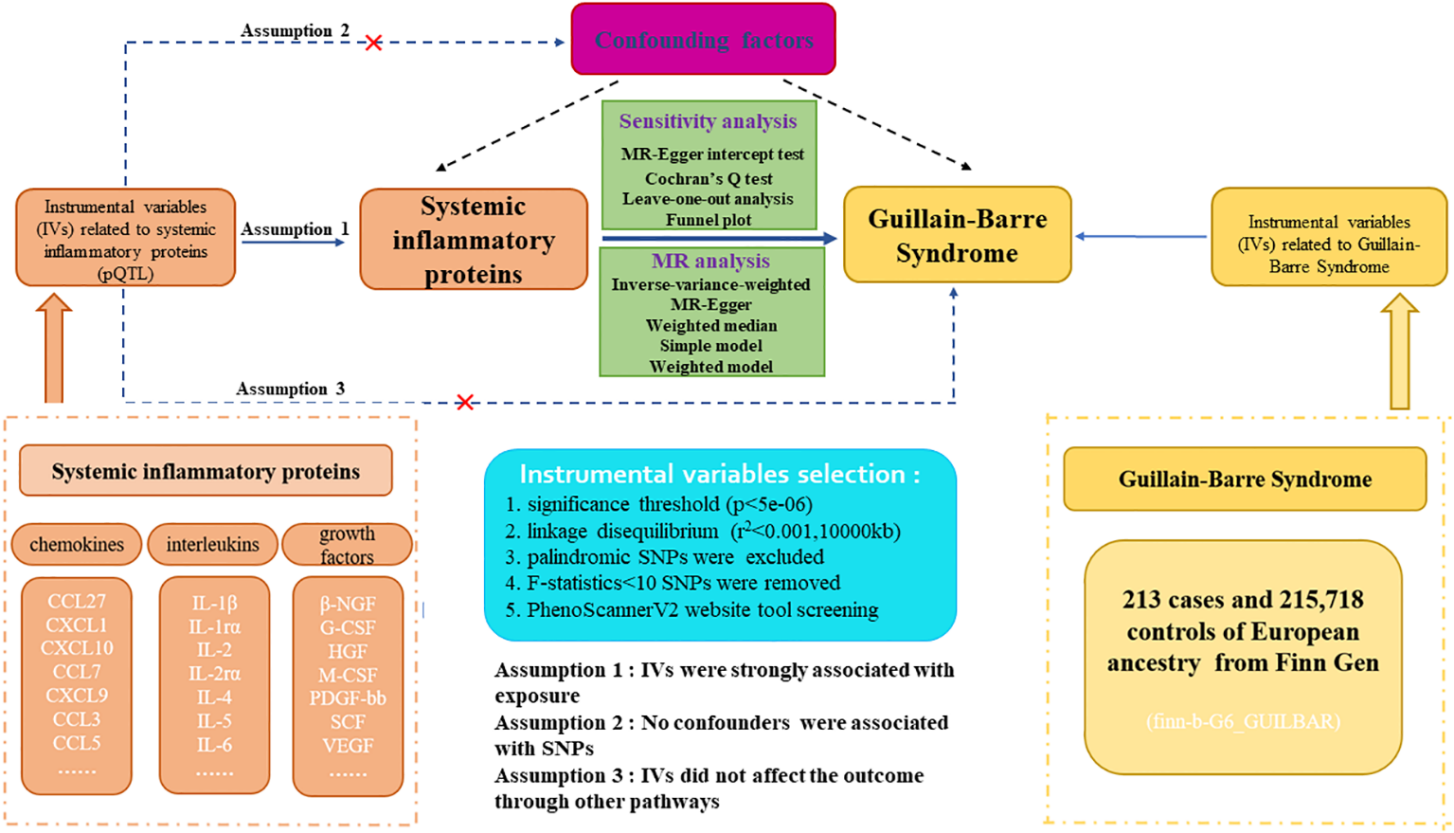
Study design and flowchart of two-sample Mendelian randomization for systemic inflammatory proteins and Guillain-Barre Syndrome. pQTL, proteins quantitative trait loci; SNP, single nucleotide polymorphism.

The flowchart and design for this study are displayed in **Figure 1**. Our research is a two-sample Mendelian-randomization (TSMR) study aimed at analyzing the causal relationship between 41 circulating inflammatory proteins and GBS utilizing genome-wide association study (GWAS) summary statistics. Detailed characteristics and information can be found in **Supplementary Table S1**. To acquire reliable results and augment the robustness of MR analysis findings, three core assumptions demand to be met when conducting TSMR analysis. First, the association between genetic variants and systemic inflammatory proteins is highly significant. Second, the SNPs are independent of potential confounding factors. Third, the instrumental variables can only influence outcomes through exposure factors, and the presence of horizontal pleiotropy is disregarded (14). Before MR analysis, we conducted the relevance assumption test and independent assumption test. We selected genetic instrumental variables for each systemic inflammatory protein to detect the causal association between every inflammatory protein and GBS. The application of publicly attainable GWAS summary datasets obviated the requirement for ethical approval and patient informed consent. All of the MR analysis and sensitivity analysis programs were conducted with the R packages of “Two Sample MR”, “fdrtool”, and “MR-PRESSO” (version 4.2.3).

### 2.2 Data sources

#### 2.2.1 Systemic inflammatory proteins

These 41 systemic inflammatory proteins database was obtained from one of the most comprehensive inflammatory proteins studies by Ahola-Olli et al. (15), encompassing the Cardiovascular Risk in Young Finns Study, FINRISK1997, and FINRISK2002. There was no overlap among these cohorts. Finally, 8,293 Finnish participants from three independent population-based cohorts were included. The mean age of the individuals was 52, and the mean BMI was 27.2kg/m^2^. The total follow-up span was from 1980 to 2011. These distributions of 41 inflammatory proteins were normalized by utilizing converse transformation. The explicit information and definition of 41 circulating inflammatory proteins were demonstrated in **Supplementary Table S2**.

#### 2.2.2 Guillain-Barre Syndrome

The summary datasets for GBS participants were obtained from the publicly attainable GWAS database, encompassing 213 cases and 215,718 controls of European descent from the Finnish independent cohort. The analysis was adjusted for both specific principal components and relevant covariates specific to each GWAS, while the overall analysis accounted for principal components. GBS individuals were diagnosed by experienced neurological physicians at each cooperating institution. The diagnosed data were investigated through gathering medical records. The detailed description of GBS GWAS datasets is exhibited in **Figure 1** and **Supplementary Table S1**.

### 2.3 Selection standards for instrumental variables

We applied comprehensive GWAS summary statistics for 41 systemic inflammatory proteins to extract instrumental variables through a series of control steps. The criteria for eligibility included: (1) given the scarcity of SNPs reaching genome-wide significance, we widened the cutoff value to P<5e-06 to extract qualified instrumental variables, consistent with Z. Song. et al.’s study (16). (2) the influence of robust linkage disequilibrium (LD) between SNPs was eliminated by employing an LD cutoff for the extracted SNPs (r²<0.001, distance>10,000kb), assuring SNPs should be independently associated with the exposure (17). (3) intermediate allele frequency palindromic SNPs were removed due to allele frequencies lacking in the GWAS data of systemic inflammatory proteins. (4) to remove bias arising from weak instrumental variables, we calculated the F-statistics for each SNP to assess statistical strength. The F-statistic was used to assess the strength of each SNP as an instrumental variable, with a value exceeding 10 indicating a robust instrument (18). Furthermore, for each exposure, we harmonized instrumental variables to assure compatibility and consistency between diverse data sources and variables. The brief selection criteria of genetic instrumental variables in this study are shown in **Figure 1**.

### 2.4 Statistical analysis

To estimate the causal effects of systemic inflammatory proteins linked to the risk of GBS by uniting various SNPs, we conducted a two-sample Mendelian randomization analysis utilizing five analysis models. The primary approach with the highly statistical power is the inverse-variance weighted (IVW) model which considers all genetic variants to be valid instrumental variables (19). This approach was regarded as the dominant approach in assessing the potential causal relationships between genetically predicted systemic inflammatory proteins and GBS. Furthermore, additional MR analysis methods, encompassing MR-Egger, weighted median, MR pleiotropy residual sum and outlier (MR-PRESSO), simple mode, and weighted mode, were incorporated as auxiliary analysis methods to IVW to provide more robust estimates across a broader range of scenarios (20). The causal effect may be measured via the MR-Egger method if genetic alterations exhibit directional pleiotropy (21). We applied the additional analyses of MR approaches with diverse modeling assumptions and strengths (such as weighted median, simple mode, etc.) to augment the robustness and stability of the findings.

### 2.5 Sensitivity analysis

To validate and quantify the strength of each instrumental variable concerning the exposure of interest, various sensitivity analysis approaches were also required. Thus, diverse approaches encompassing MR-Egger regression, Cochran’s Q statistics, MR-PRESSO, and leave-one-out (LOO) analysis were applied to validate the robustness of the significant findings (P_IVW_<0.05). Firstly, the MR-Egger intercept test was implemented to detect the presence of horizontal pleiotropy, with statistical significance defined as P-values for the intercept being less than 0.05 (22). Secondly, the heterogeneity can be assessed by utilizing Cochran’s Q statistic. The absence of heterogeneity among SNPs was indicated by a P-value greater than 0.05 in Cochran’s Q statistics (23). Thirdly, the MR-PRESSO global test was applied to investigate potential outliers as plausible pleiotropic biases and eliminate the influence of pleiotropy by removing the specific SNPs that deviated from normality. Finally, we evaluated the overall robustness with a LOO approach to our results (24). Additionally, the MR Steiger directionality test assures that effective genetic variants can explain a greater proportion of the variance in exposure compared to the outcome. If the genetic tool fails to meet this criterion, it is determined that the genetic variant has a bidirectional effect (25). Eventually, to more accurately identify true signals rather than false positive results caused by chance, we employed FDR (False Discovery Rate) correction to reduce the false positive rate (26). The presence of inflammatory proteins with a P<0.05, although not meeting the FDR-adjusted level of significance, indicated a suggestively causal association with GBS. Therefore, underlying eligible candidate inflammatory proteins for participation in the risk of GBS were identified by the following standards: (1) P-value for the primary MR analysis was significant (P_IVW_<0.05). (2) The findings of the IVW method were consistent with other approaches in direction and amplitude (scatter plots). (3) The LOO analysis did not identify any significant influential points.

### 2.6 Confounding analysis

Although we evaluated the heterogeneity and horizontal pleiotropy of the Mendelian randomization (MR) results through multiple sensitivity analysis approaches to explore any SNPs that violated Assumptions 2 and 3 of MR (**Figure 1**), there may also be little residual confounding instrumental variables. Therefore, to fulfill the independent assumption of MR, SNPs associated with confounders, encompassing blood pressure, body mass index (BMI), and smoking, were excluded by PhenoScannerV2 online tool (http://www.phenoscanner.medschl.cam.ac.uk/) screening, which was an important mean identifying confounding factors. If any SNP was found to be associated with these confounding factors (P<5e-06), MR analysis would be re-conducted after excluding this SNP to ensure the robustness of the findings.

### 2.7 Mapping candidate SNPs to genes and protein-protein interaction network analysis

We used the SNP-nexus website platform (http://www.snp-nexus.org/v4/) to perform functional annotation of SNPs associated with eligible inflammatory proteins by mapping each queried variant to its closest gene (27). Additionally, we visualized and predicted molecular interaction and PPI network utilizing STRING and GeneMANIA database. The degree algorithm of STRING software was employed to rank the significant proteins in PPI networks. GeneMANIA database can provide a protein-protein interactive network on gene and protein pathways, co-localization, co-expression, and functional assays with pinpoint accuracy of prediction algorithm (28).

### 2.8 Single-cell RNA sequencing analysis

We gained the single-cell RNA-sequencing (scRNA-seq) data of neuroepithelium tissue from the Panglao DB database (https://panglaodb.se/), a user-centric single-cell sequencing dataset for the scientific community that focused on single-cell RNA sequencing tests from mice and humans (29). We utilized the “Sample” module to search the datasets. “Human” and “Tissue” were set as screening conditions. The “Seurat” R package was employed to analyze the scRNA-seq data (30). This database encompassed 1368 single-cell RNA-sequencing dataset samples. The publicly attainable datasets utilized in the present study had acquired the necessary ethical approvals.

### 2.9 Molecular docking technology and potential therapeutic drugs prediction

With the RCSD Protein Data Bank (http://www.pdb.org), we acquired the crystal structures of targets (31). PyMOL software was utilized to isolate the primary ligand from the target protein and eliminate extraneous water molecules, phosphates, and other inactive ligands associated with the target protein (32). The 3D structural files of ligands were obtained from TCMSP, and with Chem3D software, their energy was minimized. The protein and active components were stored in the *pdb* format, while the AutoDockTools 1.5.6 software was employed to convert the *pdb* format of both the active components and target proteins into the *pdbqt* format. The parameters for the active pocket were configured, and AutoDock was employed for docking. The active component was deemed to exhibit favorable target binding activity when the binding energy reached or fell below -5.0kJ/mol (33). Discovery Studio 2019 was utilized to visualize the docking results, thereby predicting the potential drug targets.

## 3 Results

### 3.1 Selection of the instrumental variables

We performed a two-sample MR analysis to estimate the causal links of genetically determined inflammatory proteins on the risk of GBS. Taking into consideration the limited genetic variance, as well as the restricted number of SNPs and low statistical powers, the MR analysis was performed by widening the cutoff to P-value<5e-06. The number of identified instrumental variables for 41 circulating inflammatory proteins ranged from 3 to 22 (**Supplementary Table S2**). Furthermore, the F-statistics of all instrumental variables surpassed ten, suggesting that these instrumental variables were sufficiently valid for the MR analysis and that the weak instrumental bias was improbable to occur.

### 3.2 MR estimates and sensitivity analysis findings of genetically predicted 41 inflammatory proteins on GBS

**Figure 2:**
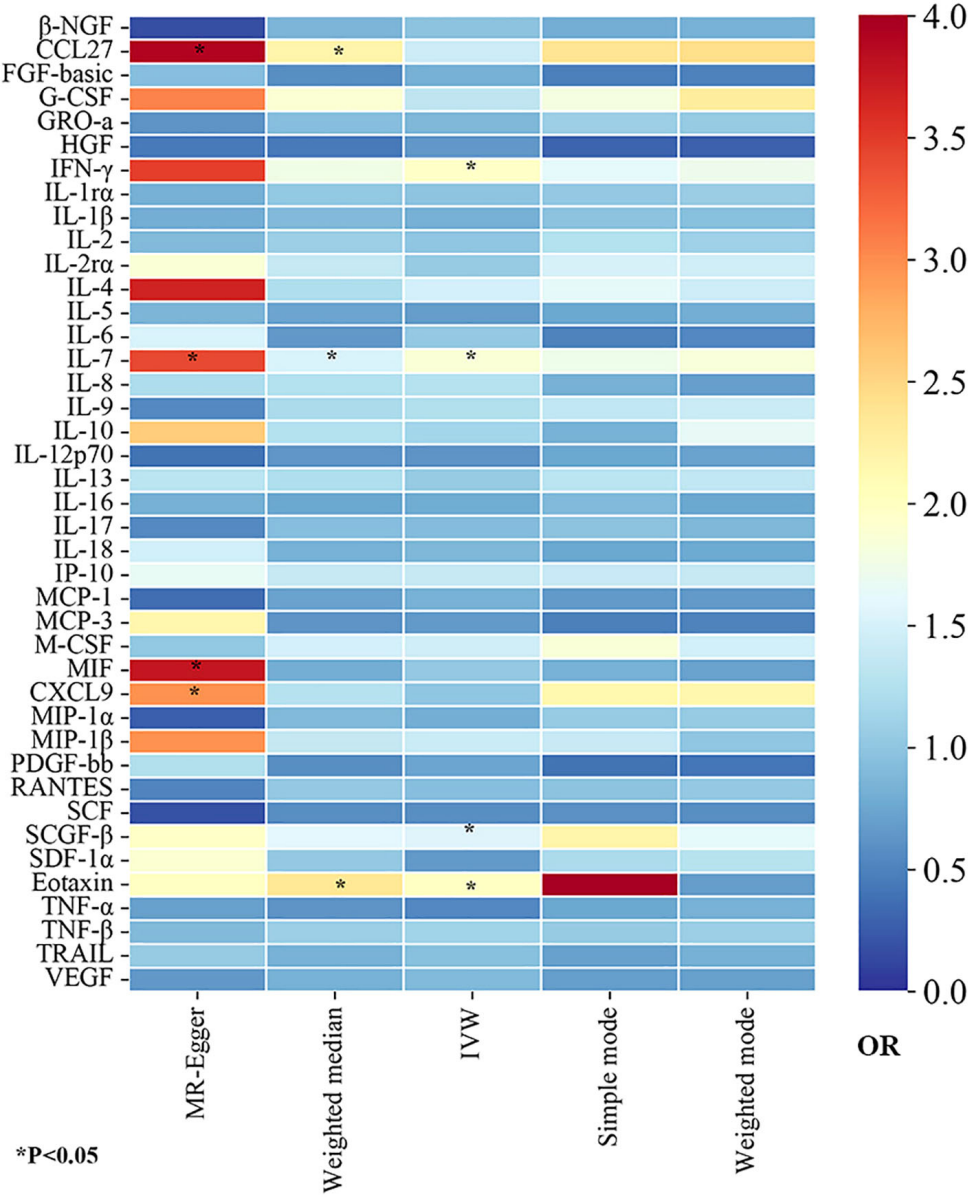
The heatmaps of five Mendelian randomization analysis methods. Different color blocks represent different odds ratio values. OR, odds ratio.

In this study, to detect the causal links of 41 inflammatory proteins on the risk of GBS, we utilized the IVW approach as the dominant method, complemented by MR-Egger, weighted mode, simple mode, and weighted median. To visually represent the strength of instrumental variables and make the compiled data more intuitive, we created heatmaps to present the estimates of the MR findings for five methods (**Figure 2**). The detailed MR estimates of diverse approaches are presented in **Supplementary Table S3**. Moreover, in order to interpret causality more rigorously, the significance threshold was adjusted according to the FDR correction. The P_IVW_-value of FDR correction was listed in **Supplementary Table S4**. In addition, due to the IVW approach being susceptible to weak instrumental bias, sensitivity analyses were implemented to assure the reliability and stability of the causality. The sensitivity analysis findings for evaluating the reliability of our MR estimates are demonstrated in **Supplementary Table S3**. Meanwhile, the findings of MR-PRESSO global test showed no outliers and horizontal pleiotropy (MR-PRESSO global test P>0.05, **Supplementary Table S4**), suggesting the robustness and credibility of the MR estimates.

### 3.3 Identification of specific inflammatory proteins linked to the risk of GBS

**Figure 3:**
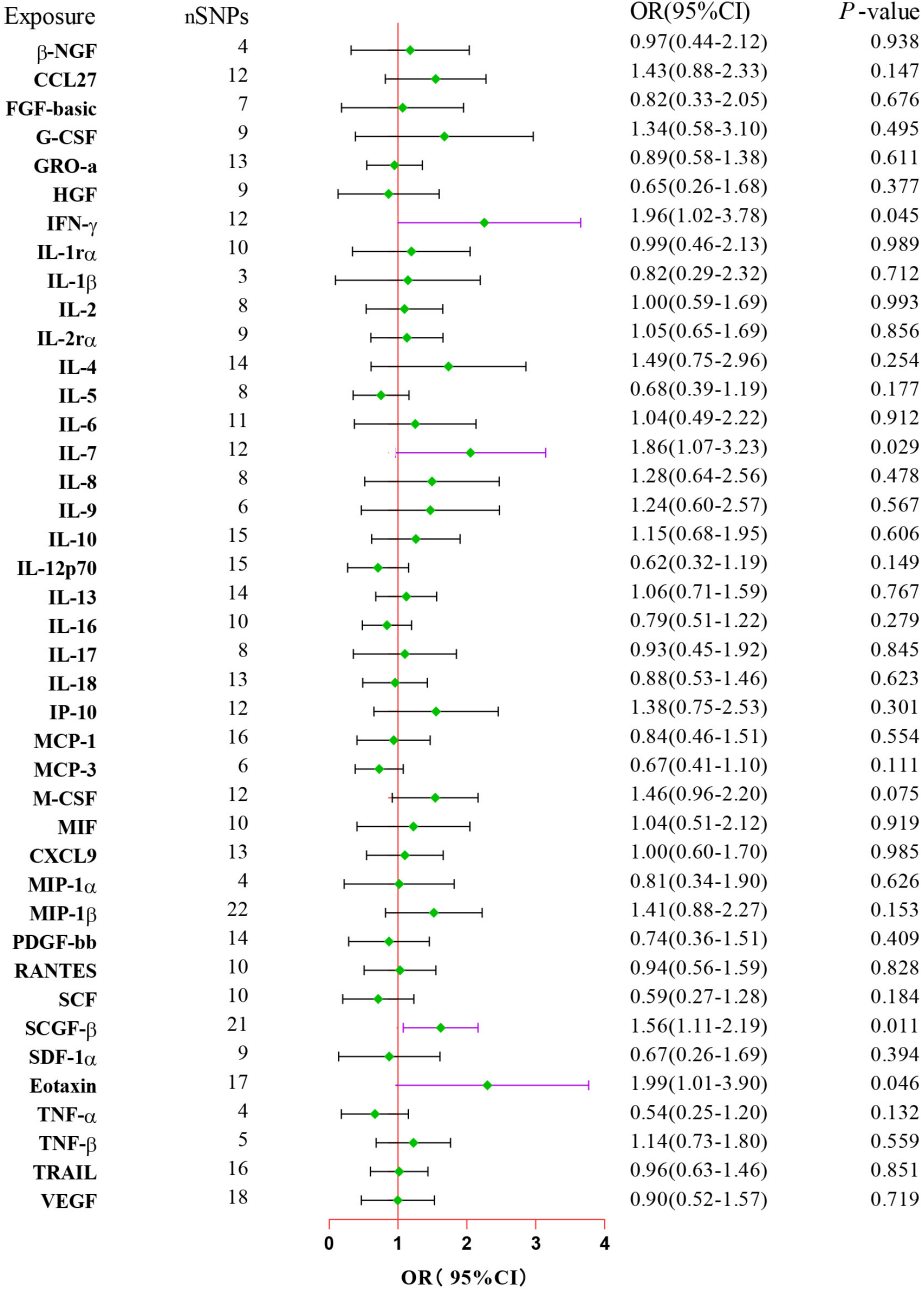
Forest plot of the MR estimates for the associations between 41 inflammatory proteins and Guillain-Barre Syndrome. The inverse variance weighted method is considered the main approach. CI, confidence interval; OR, odds ratio.

**Figure 4:**
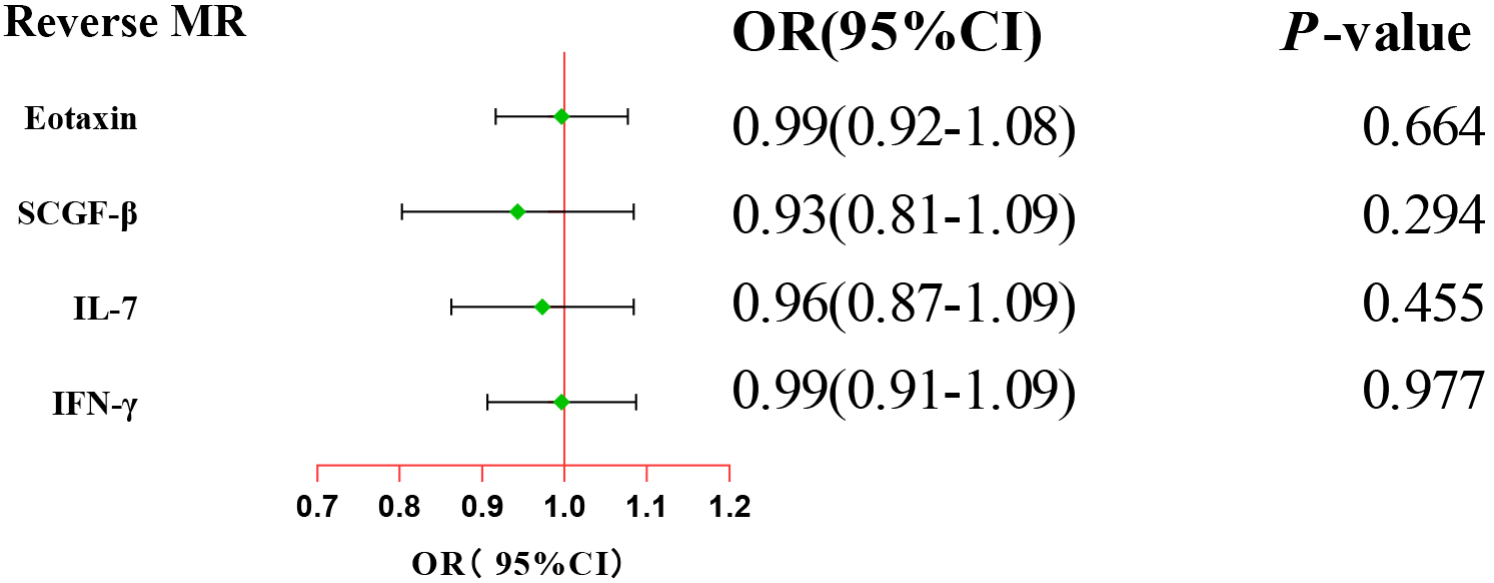
Forest plot of the reverse MR estimates. The inverse variance weighted method is considered the main approach. CI, confidence interval; OR, odds ratio.

**Figure 5:**
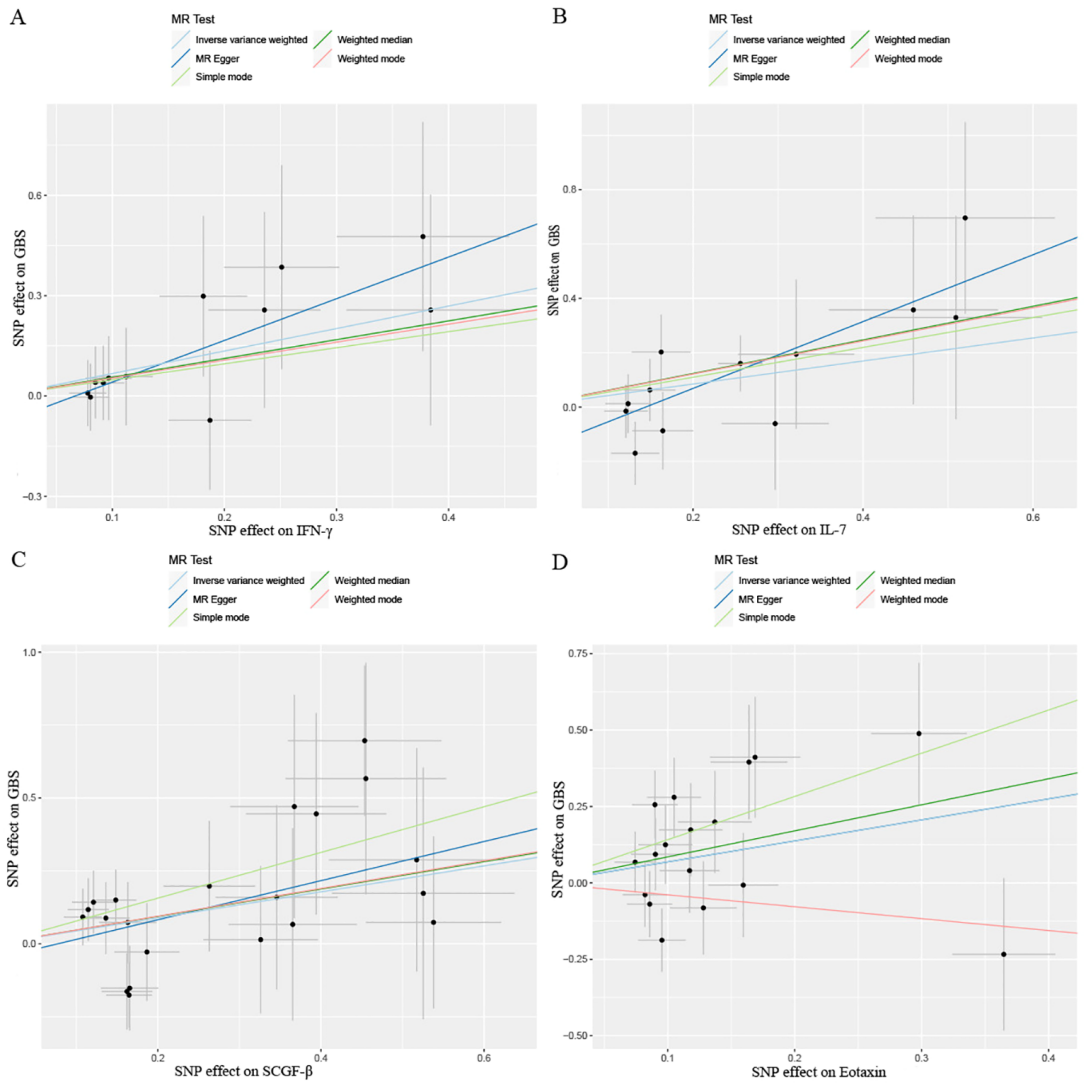
Scatter plots results of MR analysis between four identified inflammatory proteins and Guillain-Barre Syndrome. **(A)** IFN-γ, **(B)** IL-7, **(C)** SCGF-β, **(D)** Eotaxin. SNP, single nucleotide polymorphism.

**Figure 6:**
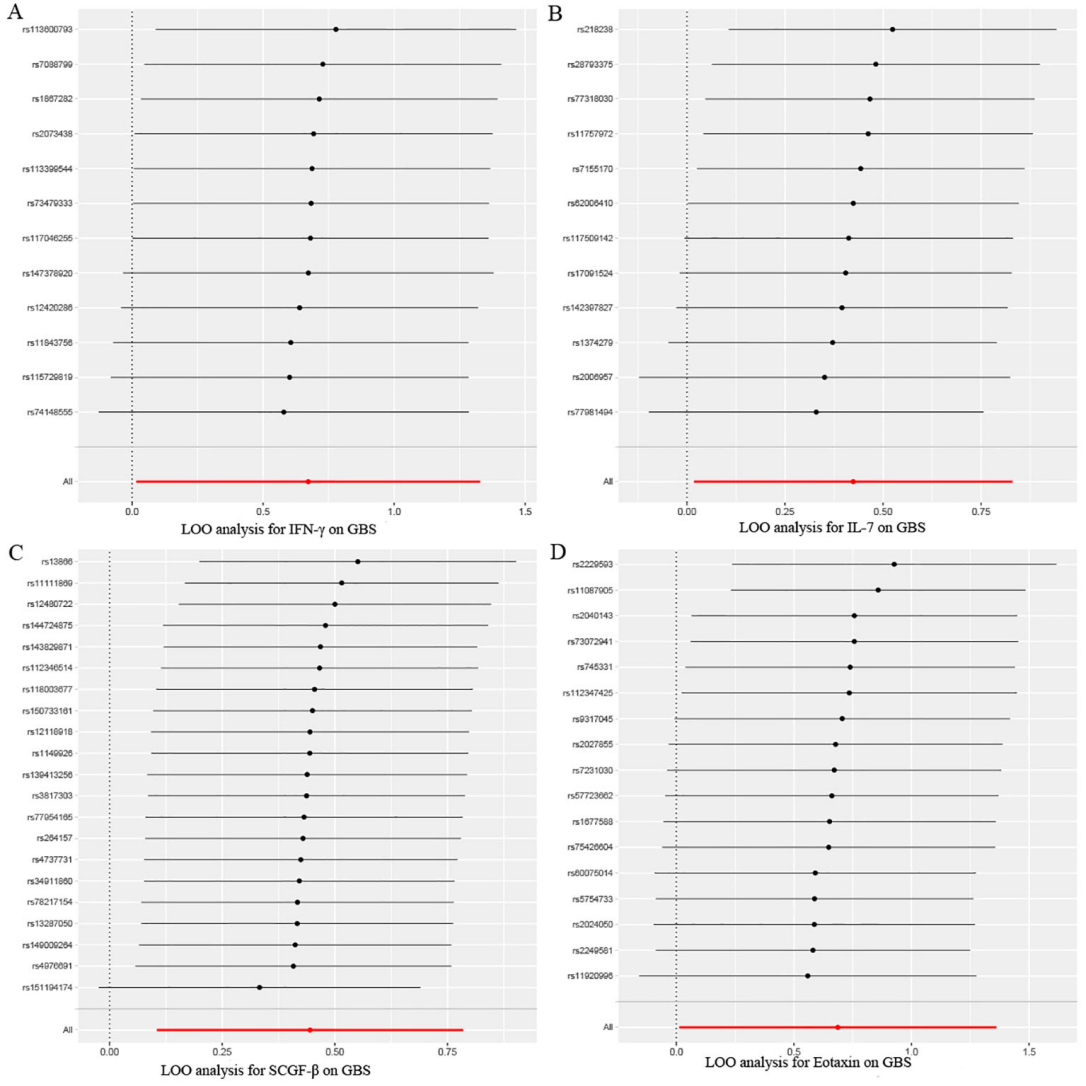
Leave-one-out sensitivity analysis results of MR analysis between four identified inflammatory proteins and Guillain-Barre Syndrome. **(A)** IFN-γ, **(B)** IL-7, **(C)** SCGF-β, **(D)** Eotaxin.

After combining complementary and sensitivity analyses, four eligible inflammatory proteins that met the rigorous screening standards were identified as the candidate inflammatory proteins linked to the risk of GBS. Specifically, as shown in **Figure 3**, IFN-γ (OR:1.96, 95%CI: 1.02-3.78, P_IVW_=0.045), IL-7 (OR:1.86, 95%CI: 1.07-3.23, P_IVW_=0.029), SCGF-β (OR:1.56, 95%CI: 1.11-2.19, P_IVW_=0.011), and Eotaxin (OR:1.99, 95%CI: 1.01-3.90, P_IVW_=0.046) were involved in the occurrence and development of GBS. These four cytokines suggested potential causal associations with an elevated risk of GBS. However, after the FDR correction test, they lost the causal associations with GBS. In short, our analysis identified four possible risky factors related to GBS. Furthermore, to augment the reliability and robustness of the findings and improve the interpretability of the study results, we performed reverse MR analysis between genetically predicted GBS and 41 inflammatory proteins. The MR estimates of GBS on systemic inflammatory proteins were displayed in **Figure 4** and **Supplementary Table S5**, indicating that genetically determined GBS showed no causal association with systemic inflammatory proteins. Additionally, scatter plots across various tests of links of four genetically determined inflammatory proteins with the risk of GBS were shown in **Figures 5A–D**. The findings of LOO analysis similarly manifested that no single SNP had a substantial impact on the stability of analysis outcomes (**Figures 6A–D**). Additionally, **Table 1** presented the sensitivity analysis findings, suggesting the reliability and robustness of our MR analysis results (all P-value>0.05). Moreover, as shown in the funnel plots (**Supplementary Figure S1**), the distribution of SNPs was symmetrical, indicating that estimates were not violated.

**Table 1:** Horizontal pleiotropy and heterogeneity analysis for the four identified inflammatory proteins.

Inflammatory proteins	Subcategory	Pleiotropy		Heterogeneity			
		MR-Egger Intercept test		MR-Egger		IVW	
		Intercept	*P-*value	Q-statistic	*P-*value	Q-statistic	*P-*value
IFN-γ	Others	-0.0833	0.357	2.173	0.995	3.106	0.989
IL-7	Interleukins	-0.1774	0.095	6.134	0.804	9.543	0.572
SCGF-β	Growth factors	-0.0517	0.486	19.132	0.448	19.639	0.481
Eotaxin	Chemokines	-0.00017	0.999	26.471	0.053	26.469	0.068

### 3.4 Confounding analysis and protein-protein interactive network

**Figure 7:**
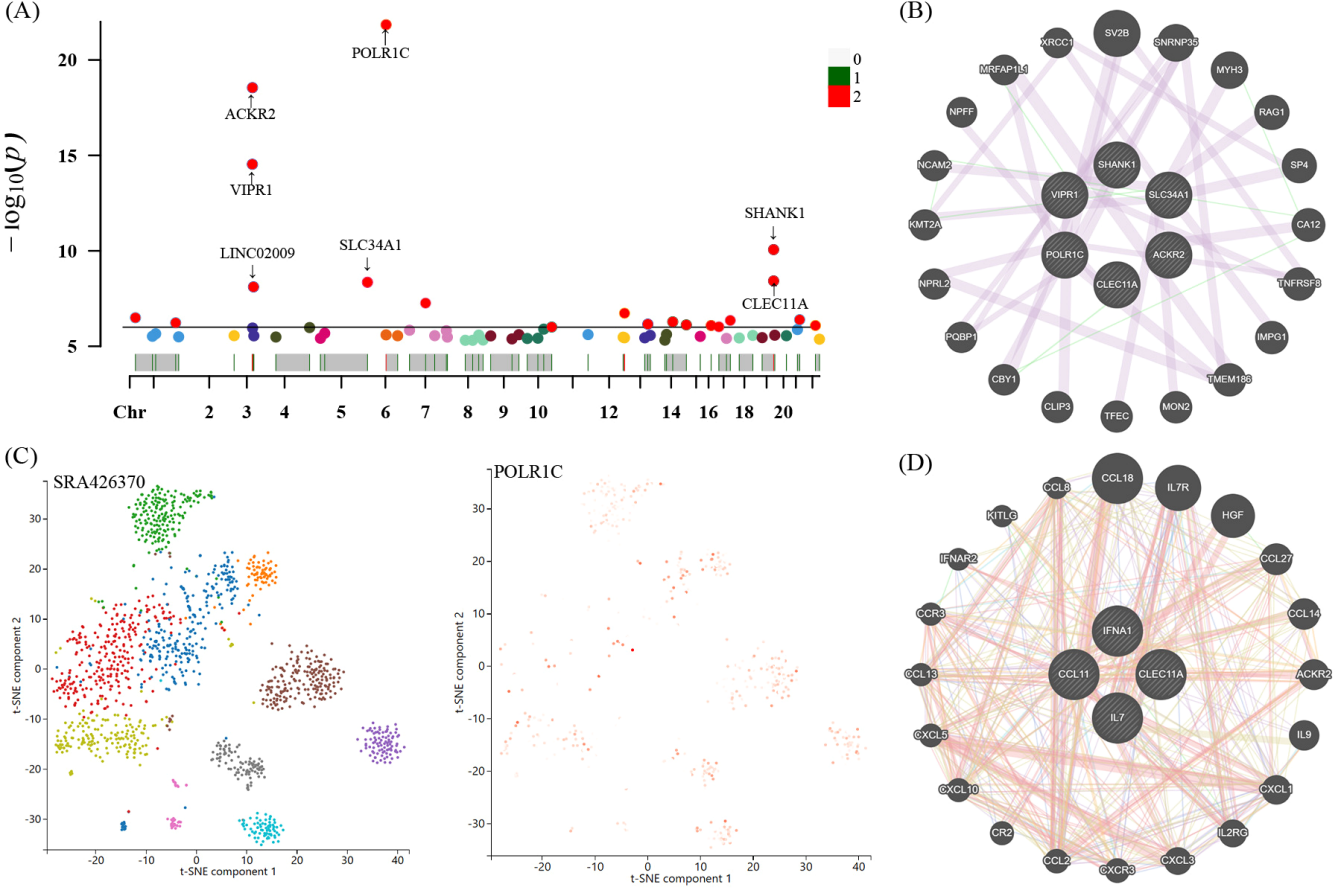
Manhattan plot exhibited the traits of genetic instrumental variables associated with four candidate inflammatory proteins and significant genes were labelled **(A)**. The most significant gene POLR1C expressed levels and distribution in neuroepithelium tissues through single-cell RNA sequencing analysis **(C)**. Protein-protein interactive (PPI) network by using GeneMANIA platform **(B, D)**. Chr, chromosome.

For these four candidate inflammatory proteins, we further manually detected these protein quantity trait loci (pQTL) for the second common trait (alcohol consumption, smoking, BMI, and blood pressure). Looking closely at the PhenoScannerV2 online platform (http://www.phenoscanner.medschl.cam.ac.uk/), we discovered that 41 pQTL were not related to any confounding factors. The corresponding gene information of instrumental variables unraveling these four inflammatory proteins linked to the risk of GBS were shown in **Supplementary Table S6**. The Manhattan plot exhibited the distribution of genetic locus associated with four identified inflammatory proteins and significant genes were labelled in **Figure 7A**. Briefly, Manhattan plots revealed that the most significant genes, encompassing POLR1C, ACKR2, VIPR1, SLC34A1, SHANK1, and CLEC11A, were primarily connected with the modulation and function of target genes associated with IFN-γ, IL-7, SCGF-β, and Eotaxin. Moreover, we applied the GeneMANIA online tool to establish and visualize a protein-protein interactive (PPI) network of overlapping hub genes (**Figure 7B**).

### 3.5 Single-cell RNA sequencing analysis results

Single-cell sequencing technology provided cell-specific genetic information and revealed the detailed function and role of target genes. We analyzed scRNA-seq data of the neuroepithelium sample SRA426370 from the Panglao DB database along with cell clustering results and cell type information. After subjecting the data to meticulous quality control procedures, we employed the t-SNE technique to visualize the high-dimensional scRNA-seq data. Data downloaded from Panglao DB demonstrated that the most significant gene POLR1C was expressed in neuroepithelium tissue to varying degrees. As demonstrated in **Figure 7C**, neuroepithelium cells were segmented into diverse cell clusters, and the gene POLR1C was designated as the overlay expression. Therefore, we might infer that POLR1C may played a vital role in the biological process of GBS, which aligned with the results of the Manhattan plot exhibition (**Figure 7A**).

### 3.6 Molecular docking results

**Figure 8:**
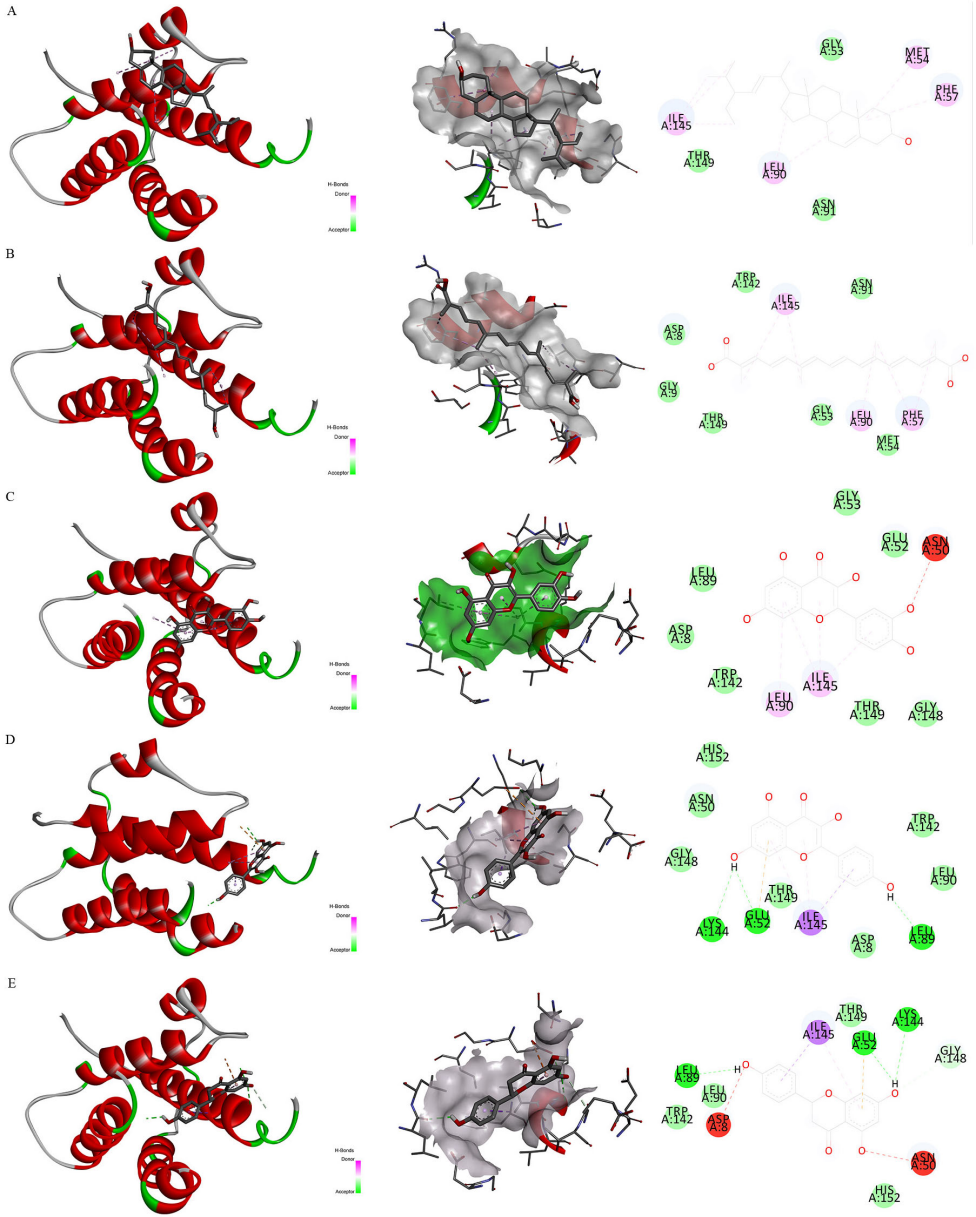
Molecular docking. Note. Binding mode of proteins and ligands. **(A)** Binding mode of IL-7 with stigmasterol; **(B)** Binding mode of IL-7 with saffronin; **(C)** Binding mode of IL-7 with quercetin; **(D)** Binding mode of IL-7 with kaempferol; **(E)** Binding mode of IL-7 with naringenin.

Firstly, **Figure 7D** exhibited the protein-protein interactive network of four candidate target proteins. Secondly, based on the Protein Data Bank (PDB) database, we screened several anti-inflammatory or neuroprotective small molecular compounds, encompassing stigmasterol, saffronin, quercetin, kaempferol, and naringenin. These compounds were selected as the key active ingredients to conduct molecular docking with IL-7. The key components and hub protein were validated by molecular docking utilizing the binding energy that reached or fell below -5.0 kcal/mol as the criterion (34). Finally, the binding energy ranged from -6.4 to -5.1 kcal/mol, and the RMSD was less than 2, suggesting that the significant active ingredients of anti-inflammatory or neuroprotective small molecular compounds were well with the target proteins. This suggests that the findings of our study are reliable, as shown in **Figure 8** and **Supplementary Table S7**.

## 4 Discussion

In this study, we elucidated the causal effects between 41 genetically determined circulating inflammatory proteins and the risk of GBS by using genetic variation as instrumental variables in a two-sample Mendelian randomization analysis. Through strict screening standards and extensive sensitivity analysis methods, four candidate inflammatory proteins (IFN-γ, IL-7, SCGF-β, and Eotaxin) were identified as the significant inflammatory cytokines causally associated with the occurrence and progression of GBS. Furthermore, we employed the Panglao DB database to conduct the single-cell RNA sequencing analysis and discovered significant genes, encompassing POLR1C, ACKR2, VIPR1, SLC34A1, SHANK1, and CLEC11A, were enriched in diverse cell clusters of neuroepithelium tissue. Meanwhile, for the candidate key proteins, we utilized molecular docking techniques to identify several anti-inflammatory or neuroprotective small molecular compounds, encompassing stigmasterol, saffronin, quercetin, kaempferol, and naringenin.

In the present study, our findings manifest that genetically predicted circulating level of IFN-γ has a causally positive association with the risk of GBS. By coincidence, a previous study conducted by H. L. Zhang et al. revealed that IFN-γ facilitated the progression of GBS, which aligned with our results (35). Interestingly, in the research, they also showed that the cytokine IFN-γ played a pivotal role in Th-1-mediated autoimmune disorders by directing the immune response towards a Th1 phenotype through promoting T cell differentiation and inhibiting the development of Th2 cells, as observed in autoimmune disorders like GBS (35). Additionally, they presented a comprehensive overview of the current understanding regarding the inflammatory and immunoregulatory role of IFN-γ in GBS and experimental autoimmune neuritis (EAN), indicating that targeting IFN-γ could be an underlying therapeutic strategy for GBS (35). However, another experimental study performed by R. Press et al. demonstrated that pretreatment levels of blood mononuclear cells (MNC) spontaneously secreting IL-10 were found to be higher in the acute phase of GBS compared to control patients with aseptic meningitis, other neurological disorders, diabetic neuropathy, and healthy individuals (36). The levels of IFN-γ-secreting blood MNC did not show any increase throughout the course of GBS. They concluded that the involvement of IFN-γ in GBS pathogenesis was found to be negligible, while the up-regulation of IL-10 during the early phase of GBS and its association with axonal damage suggested a potential role in the pathogenetic of GBS (36). To sum up, multiple studies have shown different or even opposite results on whether IFN-γ is involved in the biological process of GBS both in laboratory settings and in living organisms. In particular, our findings suggest that genetically determined IFN-γ (OR:1.96, 95%CI: 1.02-3.78, P_IVW_=0.045) is the most significant risk factor for GBS. Hence, given the ambiguity and inconsistency of these results, further studies are required to elucidate the accurate biological mechanism and validate whether IFN-γ could be treated as a promising therapeutic target of GBS in the future.

Eotaxin, also called CCL11, is a member of the eotaxin family, which is a chemotactic agent for eosinophils that plays a role in innate immunity (37). A previous study conducted by P. Lavandoski et al. demonstrated that CCL11 has the capability to induce cellular senescence and sustain a pro-inflammatory response in human embryonic fibroblasts through the promotion of oxidative stress-induced DNA damage. Furthermore, they reported that the signaling pathway of CCL11 might play a crucial role in establishing a pro-aging environment within the lungs of asthmatic patients (38). Meanwhile, another similar research performed by D. Wu et al. showed that CCL11 played a pivotal role in facilitating the migration of eosinophils to the lungs of individuals with asthma (39). Nevertheless, to date, the association between CCL11 and neurological disorders has been poorly reported. Previous studies performed by Y. Aso et al. confirmed that serum CCL11 was elevated in patients with diabetic sensorimotor polyneuropathy (DSPN) and was associated with peripheral nerve dysfunction, in particular sensory nerve conduction velocity, suggesting that serum CCL11 may be a potential biomarker for DSPN (40). In addition, another research reported by D. Nazarinia et al. manifested that eotaxins may exert both physiological and pathological functions within the central nerve system, while CCL11 can induce neuronal cytotoxic effects by stimulating reactive oxygen species (ROS) production in microglia cells (41). CCL11 plays a vital role in psychosomatic and neuroinflammation, and the plasma levels of CCL11 are elevated in conditions characterized by neuroinflammation and neurodegenerative diseases (41). Disappointingly, the epidemiological evidence supporting the links between CCL11 and GBS was few resulting from its reliance on a case-control study design and constrained by small sample sizes. In the current study, we found that genetically predicted circulating level of CCL11 was causally associated with an elevated risk of GBS (OR:1.99, 95%CI: 1.01-3.90, P_IVW_=0.046), suggesting that further exploration may be needed to validate the findings of observational studies and investigate the accurate biological mechanism for CCL11 on GBS in the future.

IL-7, also referred to as lymphopoietin, belongs to the IL-2 family cytokines (42). IL-7 is secreted by stromal cells in the bone marrow and thymus of primary lymphoid tissues, as well as epithelial cells in the liver and gut, and endothelial cells (43). IL-7 binds to the IL-7 receptor which is composed of a high-affinity α-submit known as CD127 and the common γ-chain, playing a crucial role in the biological process of T cells development (44). Recently, there has been increasing evidence that IL-7 is involved in a multitude of autoimmune disorders, such as type I diabetes, multiple sclerosis, rheumatoid arthritis, and systemic lupus erythematosus (45). Previous studies showed that targeting components in IL-7 signaling pathways may have potential significance for treating multiple autoimmune diseases (45). As far as we know, GBS is also classified as an autoimmune-mediated peripheral neuropathy. However, we found very little research on the relationship between IL-7 and GBS by reviewing the literature. Our study findings manifested that the circulating level of IL-7 was positively linked to the risk of GBS (OR:1.86, 95%CI: 1.07-3.23, P_IVW_=0.029). In the future, in order to elucidate the precise mechanisms between IL-7 and GBS, we are expected to conduct in-depth clinical and experimental studies in this field. Finally, the epidemiological evidence of observational studies for the correlation between SCGF-β and GBS was also few. In the present study, we observed that a high genetically determined serum level of SCGF-β was found to be causally linked to an elevated risk of GBS (OR:1.56, 95%CI: 1.11-2.19, P_IVW_=0.011). On the one hand, one possible explanation was that, in our study, the sample size of GBS individuals was limited, and potential confounding factors were inevitable. On the other hand, the variations of these findings may be attributed to the different choices of instrumental variables and GWAS summary data. Additionally, our research demonstrated that several significant genes, including POLR1C, ACKR2, and VIPR1, were enriched in the diverse cell clusters of neuroepithelium tissues through single-cell RNA sequencing analysis. Hence, it is expected to further validate these findings with bulk RNA analysis in the future.

Our study possessed several advantages. Firstly, the main strength of our study is that it is the first to utilize the MR analysis approach as well as single-cell RNA sequencing analysis to systematically evaluate the causal associations between 41 inflammatory proteins and GBS. This MR design can mitigate confounding biases often presented in observational studies and provide more robust evidence of causality between exposure and outcome. Secondly, due to unbalanced horizontal pleiotropy being a common bias that may distort the MR causal estimates, we extracted the instrumental variables of inflammatory proteins by using the most extensive and updated GWAS attainable datasets. Thirdly, we identified candidate inflammatory proteins causally linked to increased risk of GBS by multiple standards and rigorous control steps, encompassing MR-Egger intercept test, Cochran’s Q statistics, MR-PRESSO, and LOO sensitivity analysis. The sensitivity analysis revealed no horizontal pleiotropy and heterogeneity, suggesting the robustness and credibility of the MR estimates. Furthermore, we employed molecular docking technology to predict underlying candidate drug targets for the treatment of GBS from genetic insights.

Nevertheless, there still exist several limitations in the present study to be considered when interpreting our findings. Initially, the extraction of instrumental variables was conducted by utilizing a widened cutoff (p<5e-06), and this may cause results bias and false-positive variants. Similarly, a previous study has also utilized the identical cutoff when investigating the causal links between 41 circulating cytokines and the risk of ankylosing spondylitis (46). Secondly, the outcome database was only from the FinnGen data and the participants of GWAS only contained European descent. Due to the distinction in genetic backgrounds and living environments, the findings of our study should be cautiously extrapolated to other populations. Thus, additional research involving various ethnic populations is essential to validate our results. Thirdly, despite performing multiple sensitivity analyses to validate MR assumptions, we cannot entirely eliminate the impact of horizontal pleiotropy and heterogeneity. Fourthly, owing to the effects of different circulating inflammatory proteins on the body that may exhibit complicated interaction during the development and evolvement of disease, performing an advanced algorithm for MR study is expected to untangle the complex interaction network of inflammatory proteins. Additionally, our study is restricted to public databases and lacks real-world clinical research, suggesting that these potential links are required to be validated through conducting further experimental and clinical research.

## 5 Conclusions

In conclusion, we explored the associations between 41 inflammatory proteins and GBS utilizing MR analysis. Our study established suggestively causal links between several specific inflammatory proteins (IFN-γ, IL-7, SCGF-β, and Eotaxin) and the risk of GBS. In particular, we utilized molecular docking technology to predict underlying candidate drug targets. In the future, further validation is warranted to assess the potential of these inflammatory proteins as therapeutic targets or biomarkers for GBS.

## Data Availability

The RNA-seq data presented in this study are available on the link (https://www.ncbi.nlm.nih.gov/Traces/study/?query_key=1&WebEnv=MCID_66d5b9081ba3e568a3bf9dc5&o=acc_s%3Aa). The GWAS dataset for Guillain-Barre Syndrome is obtained from the Open GWAS Project (http://gwas.mrcieu.ac.uk/). Further inquiries can be directed to the corresponding author.
