# Supplementary material for: Causal effects of systemic inflammatory proteins on Guillain-Barre Syndrome: insights from genome-wide Mendelian randomization, single-cell RNA sequencing analysis, and network pharmacology
Source: Front Immunol. 2024 Sep 9;15:1456663. doi: 10.3389/fimmu.2024.1456663 (PMC11416972; doi:10.3389/fimmu.2024.1456663)

**Figure S1.** The funnel plots demonstrated the distribution of four candidate inflammatory proteins’ instrumental variables. (A) IFN-γ, (B) IL-7, (C) SCGF-β, and (D) Eotaxin.


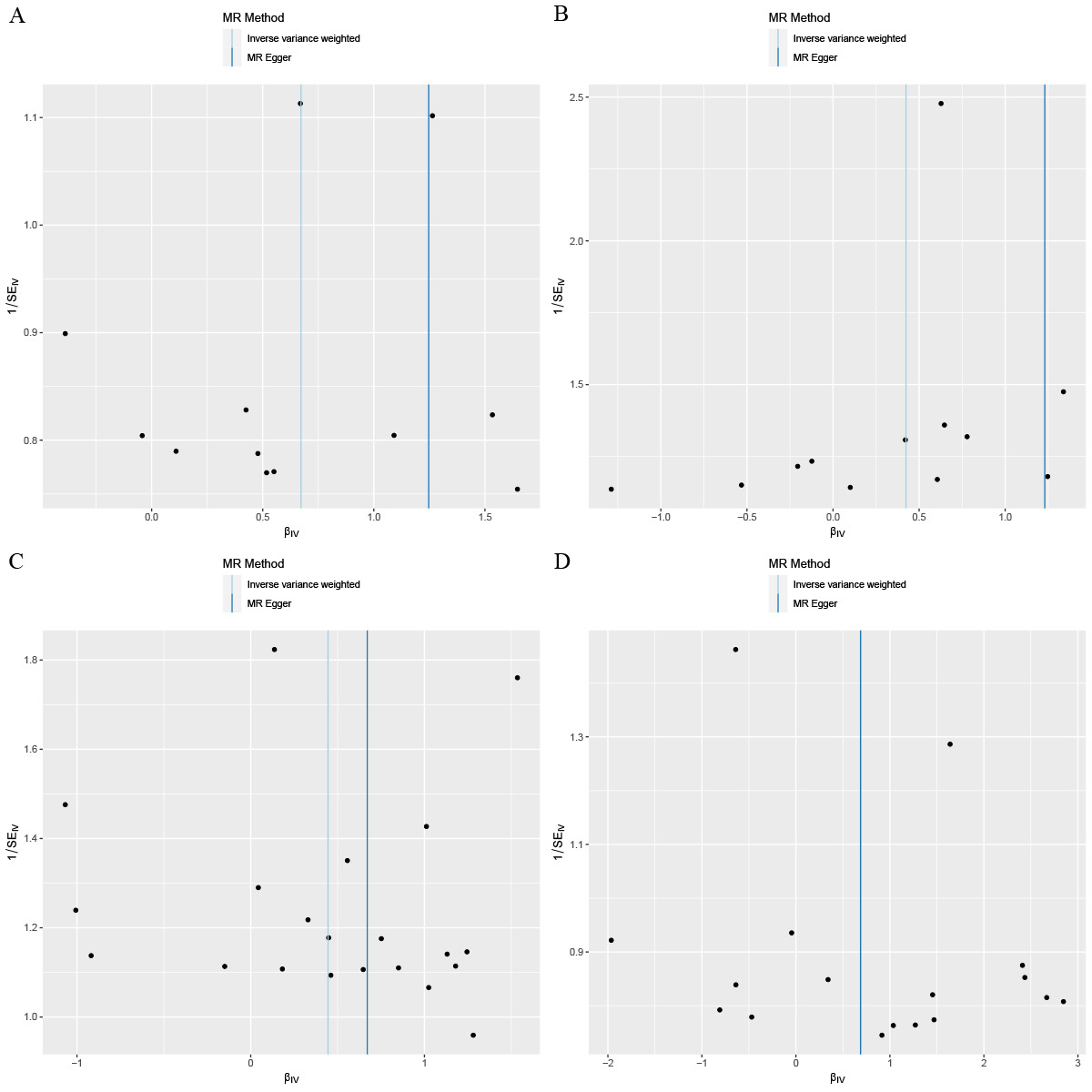

Supplement: Supplementary file 1 [file DataSheet1.zip › Supplementary materials/Supplementary Figure S1.docx]
