# Supplementary material for: Causal effects of systemic inflammatory proteins on Guillain-Barre Syndrome: insights from genome-wide Mendelian randomization, single-cell RNA sequencing analysis, and network pharmacology
Source: Front Immunol. 2024 Sep 9;15:1456663. doi: 10.3389/fimmu.2024.1456663 (PMC11416972; doi:10.3389/fimmu.2024.1456663)
Supplement: Supplementary file 1 [file DataSheet1.zip › Supplementary materials/Supplementary Table S1.docx]

**Table S1.** Brief characteristics description of 41 systemic inflammatory proteins and Guillain-Barre Syndrome GWAS cohorts involved in this study.

| **Exposure or outcome** | **Source** | **Sample size** | **Ancestry** | **Access Link** | **PMID** |
| --- | --- | --- | --- | --- | --- |
| 41 systemic inflammatory proteins | Finn Gen | 8,293 participants | European | http://computationalmedicine.fi/data#  Cytokine_GWAS | 27989323 |
| Guillain-Barre Syndrome | Finn Gen | 213 cases and 215,718controls | European | http://gwas.mrcieu.ac.uk/datasets/ finn-b-G6_GUILBAR/ | / |
