# Supplementary material for: Causal effects of systemic inflammatory proteins on Guillain-Barre Syndrome: insights from genome-wide Mendelian randomization, single-cell RNA sequencing analysis, and network pharmacology
Source: Front Immunol. 2024 Sep 9;15:1456663. doi: 10.3389/fimmu.2024.1456663 (PMC11416972; doi:10.3389/fimmu.2024.1456663)
Supplement: Supplementary file 1 [file DataSheet1.zip › Supplementary materials/Supplementary Table S2.docx]

**Table S2.** Details of the number of genetic IVs and *F*-statistic for each inflammatory protein. ***(P* < 5 ×10^-6^).**

| Inflammatory proteins | Abbreviations | No. of SNPs | *F*-statistic (range) | Number |
| --- | --- | --- | --- | --- |
| Cutaneous T-cell attracting (CCL27) | CTACK | 12 | 22.92 (20.84-31.44) | GCST004420 |
| Beta nerve growth factor | β-NGF | 4 | 24.59 (20.83-35.43) | GCST004421 |
| Vascular endothelial growth factor | VEGF | 18 | 30.26 (20.88-71.72) | GCST004422 |
| Macrophage migration inhibitory factor (glycosylation-inhibiting factor) | MIF | 10 | 23.15 (21.07-27.07) | GCST004423 |
| TNF-related apoptosis inducing ligand | TRAIL | 16 | 52.36 (20.83-345.00) | GCST004424 |
| Tumor necrosis factor-beta | TNF-β | 5 | 25.31 (20.98-38.27) | GCST004425 |
| Tumor necrosis factor-alpha | TNF-α | 4 | 22.46 (21.74-23.54) | GCST004426 |
| Stromal cell-derived factor-1 alpha (CXCL12) | SDF-1α | 9 | 22.46 (20.84-27.84) | GCST004427 |
| Stem cell growth factor beta | SCGF-β | 21 | 24.15 (20.83-42.11) | GCST004428 |
| Stem cell factor | SCF | 10 | 22.99 (21.13-25.82) | GCST004429 |
| Interleukin-16 | IL-16 | 10 | 28.99 (21.21-53.45) | GCST004430 |
| Regulated on activation, normal T Cell expressed and secreted (CCL5) | RANTES | 10 | 22.80 (20.84-27.21) | GCST004431 |
| Platelet derived growth factor BB | PDGF-bb | 14 | 24.24 (21.15-31.12) | GCST004432 |
| Macrophage inflammatory protein-1β (CCL4) | MIP-1β | 22 | 28.33 (20.95-87.99) | GCST004433 |
| Macrophage inflammatory protein-1α (CCL3) | MIP-1α | 4 | 22.04 (20.94-24.11) | GCST004434 |
| Monokine induced by interferon-gamma (CXCL9) | MIG | 13 | 23.71 (21.04-30.58) | GCST004435 |
| Macrophage colony-stimulating factor | M-CSF | 12 | 21.97 (20.92-25.52) | GCST004436 |
| Monocyte specific chemokine 3 (CCL7) | MCP-3 | 6 | 22.22 (20.77-26.39) | GCST004437 |
| Monocyte chemotactic protein-1 (CCL2) | MCP-1 | 16 | 25.27 (21.01-41.97) | GCST004438 |
| Interleukin-12p70 | IL-12p70 | 15 | 24.48 (20.89-48.69) | GCST004439 |
| Interferon gamma-induced protein 10 (CXCL10) | IP-10 | 12 | 22.27 (20.87-24.72) | GCST004440 |
| Interleukin-18 | IL-18 | 13 | 23.41 (21.29-27.31) | GCST004441 |
| Interleukin-17 | IL-17 | 8 | 23.85 (20.90-32.13) | GCST004442 |
| Interleukin-13 | IL-13 | 14 | 22.63 (20.87-25.78) | GCST004443 |
| Interleukin-10 | IL-10 | 15 | 31.34 (20.98-132.61) | GCST004444 |
| Interleukin-8 (CXCL8) | IL-8 | 8 | 21.95 (20.89-23.85) | GCST004445 |
| Interleukin-6 | IL-6 | 11 | 22.77 (20.77-30.71) | GCST004446 |
| Interleukin-1 receptor antagonist | IL-1rα | 10 | 21.94 (20.77-24.25) | GCST004447 |
| Interleukin-1-beta | IL-1β | 3 | 22.99 (21.79-25.53) | GCST004448 |
| Hepatocyte growth factor | HGF | 9 | 25.80 (21.00-55.42) | GCST004449 |
| Interleukin-9 | IL-9 | 6 | 24.40 (21.54-24.59) | GCST004450 |
| Interleukin-7 | IL-7 | 12 | 27.42 (20.84-95.19) | GCST004451 |
| Interleukin-5 | IL-5 | 8 | 22.40 (20.85-25.57) | GCST004452 |
| Interleukin-4 | IL-4 | 14 | 22.97 (20.88-26.61) | GCST004453 |
| Interleukin-2 receptor, alpha subunit | IL-2rα | 9 | 28.06 (20.93-77.94) | GCST004454 |
| Interleukin-2 | IL-2 | 8 | 22.80 (20.94-27.62) | GCST004455 |
| Interferon-gamma | IFN-γ | 12 | 23.16 (21.47-26.14) | GCST004456 |
| Growth regulated oncogene-α (CXCL1) | GRO-α | 13 | 22.97 (20.83-29.89) | GCST004457 |
| Granulocyte colony-stimulating factor | G-CSF | 9 | 22.54 (20.94-25.07) | GCST004458 |
| Basic fibroblast growth factor | bFGF | 7 | 22.04 (20.79-25.40) | GCST004459 |
| Eotaxin (CCL11) | Eotaxin | 17 | 29.38 (21.03-80.67) | GCST004460 |

Abbreviations: No., number; SNP, single nucleotide polymorphism.
