# Supplementary material for: Causal effects of systemic inflammatory proteins on Guillain-Barre Syndrome: insights from genome-wide Mendelian randomization, single-cell RNA sequencing analysis, and network pharmacology
Source: Front Immunol. 2024 Sep 9;15:1456663. doi: 10.3389/fimmu.2024.1456663 (PMC11416972; doi:10.3389/fimmu.2024.1456663)
Supplement: Supplementary file 1 [file DataSheet1.zip › Supplementary materials/Supplementary Table S3.docx]

**Table S3.** MR analysis of the associations between 41 inflammatory proteins and GBS.

| **systemic inflammatory proteins** | | **Number of SNPs** | **OR (95% CI)** | **P-value** | **P for Cochran’s Q test** | **P for MR-Egger intercept** |
| --- | --- | --- | --- | --- | --- | --- |
| IL-10 | |  |  |  |  |  |
|  | MR Egger | 15 | 2.57 (0.46 - 14.38) | 0.302 | 0.735 | 0.353 |
|  | Weighted median | 15 | 1.25 (0.60 - 2.59) | 0.545 |  |  |
|  | Inverse variance weighted | 15 | 1.15 (0.68- 1.95) | 0.606 | 0.731 |  |
|  | Simple mode | 15 | 0.83 (0.24 - 2.86) | 0.777 |  |  |
|  | Weighted mode | 15 | 1.65 (0.65- 4.15) | 0.308 |  |  |
| IL-16 | |  |  |  |  |  |
|  | MR Egger | 10 | 0.82 (0.40 - 1.68) | 0.606 | 0.511 | 0.875 |
|  | Weighted median | 10 | 0.75 (0.42- 1.34) | 0.332 |  |  |
|  | Inverse variance weighted | 10 | 0.79 (0.51- 1.22) | 0.279 | 0.609 |  |
|  | Simple mode | 10 | 0.90 (0.33- 2.45) | 0.847 |  |  |
|  | Weighted mode | 10 | 0.75 (0.36- 1.54) | 0.454 |  |  |
| IL-4 | |  |  |  |  |  |
|  | MR Egger | 14 | 3.68 (0.94 - 14.44) | 0.085 | 0.439 | 0.165 |
|  | Weighted median | 14 | 1.23 (0.48 - 3.12) | 0.664 |  |  |
|  | Inverse variance weighted | 14 | 1.49 (0.75- 2.96) | 0.254 | 0.355 |  |
|  | Simple mode | 14 | 1.63 (0.33 - 8.12) | 0.564 |  |  |
|  | Weighted mode | 14 | 1.45 (0.36- 5.79) | 0.605 |  |  |
| TNF-a | |  |  |  |  |  |
|  | MR Egger | 4 | 0.71 (0.20 -2.54) | 0.652 | 0.352 | 0.644 |
|  | Weighted median | 4 | 0.61 (0.24- 1.59) | 0.312 |  |  |
|  | Inverse variance weighted | 4 | 0.54 (0.25- 1.20) | 0.132 | 0.495 |  |
|  | Simple mode | 4 | 0.75(0.17- 3.40) | 0.739 |  |  |
|  | Weighted mode | 4 | 0.83(0.23- 3.02) | 0.790 |  |  |
| IL-7 | |  |  |  |  |  |
|  | MR Egger | 12 | 3.42 (1.33 -8.82) | 0.029 | 0.804 | 0.095 |
|  | Weighted median | 12 | 1.53 (1.02 - 2.29) | 0.041 |  |  |
|  | Inverse variance weighted | 12 | 1.86 (1.07- 3.23) | 0.029 | 0.572 |  |
|  | Simple mode | 12 | 1.73 (0.72 - 4.17) | 0.247 |  |  |
|  | Weighted mode | 12 | 1.84 (0.88 - 3.82) | 0.131 |  |  |
| IL-13 | |  |  |  |  |  |
|  | MR Egger | 14 | 1.30 (0.61 -2.75) | 0.509 | 0.809 | 0.548 |
|  | Weighted median | 14 | 1.22 (0.72 -2.08) | 0.461 |  |  |
|  | Inverse variance weighted | 14 | 1.06 (0.71 - 1.59) | 0.767 | 0.838 |  |
|  | Simple mode | 14 | 1.30 (0.53 - 3.21) | 0.572 |  |  |
|  | Weighted mode | 14 | 1.35 (0.48 - 3.75) | 0.578 |  |  |
| IL-8 | |  |  |  |  |  |
|  | MR Egger | 8 | 1.21 (0.30 - 4.80) | 0.796 | 0.121 | 0.923 |
|  | Weighted median | 8 | 1.26 (0.54 - 2.91) | 0.594 |  |  |
|  | Inverse variance weighted | 8 | 1.28 (0.64 -2.56) | 0.478 | 0.183 |  |
|  | Simple mode | 8 | 0.82 (0.18 - 3.71) | 0.799 |  |  |
|  | Weighted mode | 8 | 0.69 (0.15 - 3.25) | 0.657 |  |  |
| M-CSF | |  |  |  |  |  |
|  | MR Egger | 12 | 1.02 (0.43 -2.45) | 0.963 | 0.248 | 0.387 |
|  | Weighted median | 12 | 1.49 (0.88 -2.52) | 0.137 |  |  |
|  | Inverse variance weighted | 12 | 1.46 (0.96 -2.20) | 0.075 | 0.256 |  |
|  | Simple mode | 12 | 1.85 (0.80- 4.29) | 0.181 |  |  |
|  | Weighted mode | 12 | 1.47 (0.66 - 3.27) | 0.364 |  |  |
| IL-17 | |  |  |  |  |  |
|  | MR Egger | 8 | 0.55 (0.14 - 2.13) | 0.422 | 0.649 | 0.404 |
|  | Weighted median | 8 | 0.94 (0.34 - 2.61) | 0.906 |  |  |
|  | Inverse variance weighted | 8 | 0.93 (0.45 - 1.92) | 0.845 | 0.659 |  |
|  | Simple mode | 8 | 0.98 (0.24 - 3.97) | 0.978 |  |  |
|  | Weighted mode | 8 | 0.89 (0.29- 2.73) | 0.848 |  |  |
| IL-1ra | |  |  |  |  |  |
|  | MR Egger | 10 | 0.82 (0.09 -7.88) | 0.868 | 0.020 | 0.862 |
|  | Weighted median | 10 | 1.03 (0.48- 2.21) | 0.942 |  |  |
|  | Inverse variance weighted | 10 | 0.99 (0.46 - 2.13) | 0.989 | 0.033 |  |
|  | Simple mode | 10 | 1.04 (0.25- 4.30) | 0.961 |  |  |
|  | Weighted mode | 10 | 1.07 (0.29- 3.97) | 0.922 |  |  |
| PDGF-bb | |  |  |  |  |  |
|  | MR Egger | 14 | 1.24 (0.22 - 6.96) | 0.809 | 0.231 | 0.528 |
|  | Weighted median | 14 | 0.59 (0.24 - 1.47) | 0.257 |  |  |
|  | Inverse variance weighted | 14 | 0.74 (0.36 - 1.51) | 0.409 | 0.265 |  |
|  | Simple mode | 14 | 0.40 (0.08 - 2.03) | 0.291 |  |  |
|  | Weighted mode | 14 | 0.41 (0.08- 2.10) | 0.304 |  |  |
| IL-18 | |  |  |  |  |  |
|  | MR Egger | 13 | 1.48 (0.57 - 3.84) | 0.434 | 0.236 | 0.233 |
|  | Weighted median | 13 | 0.84 (0.45 - 1.58) | 0.323 |  |  |
|  | Inverse variance weighted | 13 | 0.88 (0.53 - 1.46) | 0.623 | 0.193 |  |
|  | Simple mode | 13 | 0.76 (0.23 - 2.53) | 0.663 |  |  |
|  | Weighted mode | 13 | 0.77 (0.27- 2.19) | 0.633 |  |  |
| G-CSF | |  |  |  |  |  |
|  | MR Egger | 9 | 3.06 (0.79 - 11.82) | 0.148 | 0.380 | 0.184 |
|  | Weighted median | 9 | 1.88 (0.69 - 5.17) | 0.219 |  |  |
|  | Inverse variance weighted | 9 | 1.34 (0.58- 3.10) | 0.495 | 0.279 |  |
|  | Simple mode | 9 | 1.81 (0.45- 7.26) | 0.429 |  |  |
|  | Weighted mode | 9 | 2.29 (0.65 - 8.04) | 0.234 |  |  |
| MCP-1 | |  |  |  |  |  |
|  | MR Egger | 16 | 0.36 (0.07 -1.82) | 0.235 | 0.696 | 0.289 |
|  | Weighted median | 16 | 0.72 (0.33 - 1.59) | 0.419 |  |  |
|  | Inverse variance weighted | 16 | 0.84 (0.46 - 1.51) | 0.554 | 0.673 |  |
|  | Simple mode | 16 | 0.67 (0.17- 2.61) | 0.573 |  |  |
|  | Weighted mode | 16 | 0.66 (0.18- 2.42) | 0.541 |  |  |
| FGF-basic | |  |  |  |  |  |
|  | MR Egger | 7 | 0.95 (0.06- 15.18) | 0.974 | 0.359 | 0.917 |
|  | Weighted median | 7 | 0.58 (0.18- 1.89) | 0.367 |  |  |
|  | Inverse variance weighted | 7 | 0.82 (0.33 - 2.05) | 0.676 | 0.481 |  |
|  | Simple mode | 7 | 0.47 (0.09 - 2.48) | 0.407 |  |  |
|  | Weighted mode | 7 | 0.49 (0.10 - 2.50) | 0.425 |  |  |
| CXCL9 | |  |  |  |  |  |
|  | MR Egger | 13 | 2.98 (1.20 - 7.39) | 0.037 | 0.527 | 0.021 |
|  | Weighted median | 13 | 1.28 (0.68 - 2.40) | 0.443 |  |  |
|  | Inverse variance weighted | 13 | 1.00 (0.60 - 1.70) | 0.985 | 0.141 |  |
|  | Simple mode | 13 | 2.15 (0.59 - 7.88) | 0.272 |  |  |
|  | Weighted mode | 13 | 2.17 (0.65 - 7.28) | 0.233 |  |  |
| HGF | |  |  |  |  |  |
|  | MR Egger | 9 | 0.45 (0.05- 4.04) | 0.497 | 0.148 | 0.716 |
|  | Weighted median | 9 | 0.45 (0.14 - 1.46) | 0.186 |  |  |
|  | Inverse variance weighted | 9 | 0.65 (0.26 - 1.68) | 0.377 | 0.201 |  |
|  | Simple mode | 9 | 0.31 (0.04 -2.33) | 0.288 |  |  |
|  | Weighted mode | 9 | 0.29 (0.06- 1.33) | 0.151 |  |  |
| TNF-b | |  |  |  |  |  |
|  | MR Egger | 5 | 0.91 (0.36 -2.29) | 0.861 | 0.809 | 0.621 |
|  | Weighted median | 5 | 1.08 (0.63 - 1.85) | 0.775 |  |  |
|  | Inverse variance weighted | 5 | 1.14 (0.73 - 1.80) | 0.559 | 0.867 |  |
|  | Simple mode | 5 | 1.06 (0.53 - 2.12) | 0.886 |  |  |
|  | Weighted mode | 5 | 1.09 (0.54 - 2.22) | 0.822 |  |  |
| VEGF | |  |  |  |  |  |
|  | MR Egger | 18 | 0.65 (0.19 - 2.16) | 0.487 | 0.149 | 0.545 |
|  | Weighted median | 18 | 0.84 (0.41- 1.70) | 0.625 |  |  |
|  | Inverse variance weighted | 18 | 0.90 (0.52 - 1.57) | 0.719 | 0.171 |  |
|  | Simple mode | 18 | 0.69 (0.18 - 2.56) | 0.581 |  |  |
|  | Weighted mode | 18 | 0.71 (0.27 - 1.88) | 0.497 |  |  |
| GRO-a | |  |  |  |  |  |
|  | MR Egger | 13 | 0.62 (0.29- 1.32) | 0.239 | 0.697 | 0.270 |
|  | Weighted median | 13 | 0.95 (0.54- 1.66) | 0.854 |  |  |
|  | Inverse variance weighted | 13 | 0.89 (0.58- 1.38) | 0.611 | 0.657 |  |
|  | Simple mode | 13 | 1.08 (0.40- 2.90) | 0.881 |  |  |
|  | Weighted mode | 13 | 1.05 (0.41 - 2.68) | 0.928 |  |  |
| IL-6 | |  |  |  |  |  |
|  | MR Egger | 11 | 1.53 (0.26 - 8.91) | 0.646 | 0.339 | 0.644 |
|  | Weighted median | 11 | 0.65 (0.24 - 1.77) | 0.399 |  |  |
|  | Inverse variance weighted | 11 | 1.04 (0.49 - 2.22) | 0.912 | 0.406 |  |
|  | Simple mode | 11 | 0.50 (0.09- 2.63) | 0.431 |  |  |
|  | Weighted mode | 11 | 0.54 (0.10 - 2.77) | 0.475 |  |  |
| SCF | |  |  |  |  |  |
|  | MR Egger | 10 | 0.19 (0.04 - 0.96) | 0.079 | 0.719 | 0.155 |
|  | Weighted median | 10 | 0.58 (0.22- 1.57) | 0.284 |  |  |
|  | Inverse variance weighted | 10 | 0.59 (0.27- 1.28) | 0.184 | 0.552 |  |
|  | Simple mode | 10 | 0.60 (0.12- 3.10) | 0.559 |  |  |
|  | Weighted mode | 10 | 0.58 (0.12 - 2.87) | 0.521 |  |  |
| Eotaxin | |  |  |  |  |  |
|  | MR Egger | 17 | 1.99 (0.36 - 10.92) | 0.441 | 0.053 | 0.999 |
|  | Weighted median | 17 | 2.35 (1.03- 5.35) | 0.043 |  |  |
|  | Inverse variance weighted | 17 | 1.99 (1.01 - 3.90) | 0.046 | 0.068 |  |
|  | Simple mode | 17 | 4.11 (0.64- 26.57) | 0.157 |  |  |
|  | Weighted mode | 17 | 0.68 (0.09 - 4.90) | 0.705 |  |  |
| IL-2 | |  |  |  |  |  |
|  | MR Egger | 8 | 0.91 (0.32- 2.58) | 0.871 | 0.839 | 0.855 |
|  | Weighted median | 8 | 1.09 (0.57 - 2.10) | 0.793 |  |  |
|  | Inverse variance weighted | 8 | 1.00 (0.59 - 1.69) | 0.993 | 0.904 |  |
|  | Simple mode | 8 | 1.26 (0.49- 3.25) | 0.649 |  |  |
|  | Weighted mode | 8 | 1.10 (0.45 - 2.69) | 0.842 |  |  |
| MIP-1α | |  |  |  |  |  |
|  | MR Egger | 4 | 0.27 (0.02 - 3.88) | 0.439 | 0.539 | 0.486 |
|  | Weighted median | 4 | 0.90 (0.32 - 2.49) | 0.836 |  |  |
|  | Inverse variance weighted | 4 | 0.81 (0.34- 1.90) | 0.626 | 0.582 |  |
|  | Simple mode | 4 | 1.06 (0.24 - 4.78) | 0.944 |  |  |
|  | Weighted mode | 4 | 1.05 (0.24 - 4.64) | 0.951 |  |  |
| IFN-γ | |  |  |  |  |  |
|  | MR Egger | 12 | 3.48 (0.91- 13.26) | 0.098 | 0.995 | 0.357 |
|  | Weighted median | 12 | 1.75 (0.74 - 4.15) | 0.202 |  |  |
|  | Inverse variance weighted | 12 | 1.96 (1.02 - 3.78) | 0.045 | 0.989 |  |
|  | Simple mode | 12 | 1.62 (0.47 - 5.62) | 0.465 |  |  |
|  | Weighted mode | 12 | 1.71 (0.52 - 5.61) | 0.395 |  |  |
| IL-9 | |  |  |  |  |  |
|  | MR Egger | 6 | 0.56 (0.09- 3.29) | 0.554 | 0.627 | 0.389 |
|  | Weighted median | 6 | 1.19 (0.44- 3.20) | 0.734 |  |  |
|  | Inverse variance weighted | 6 | 1.24 (0.60 -2.57) | 0.567 | 0.619 |  |
|  | Simple mode | 6 | 1.35 (0.34- 5.37) | 0.684 |  |  |
|  | Weighted mode | 6 | 1.42 (0.36- 5.61) | 0.636 |  |  |
| IL-5 | |  |  |  |  |  |
|  | MR Egger | 8 | 0.86 (0.26 - 2.89) | 0.815 | 0.670 | 0.689 |
|  | Weighted median | 8 | 0.74 (0.37 - 1.50) | 0.409 |  |  |
|  | Inverse variance weighted | 8 | 0.68 (0.39 - 1.19) | 0.177 | 0.754 |  |
|  | Simple mode | 8 | 0.76 (0.25 - 2.26) | 0.632 |  |  |
|  | Weighted mode | 8 | 0.80 (0.27 - 2.33) | 0.691 |  |  |
| MCP-3 | |  |  |  |  |  |
|  | MR Egger | 6 | 2.16 (0.79 - 5.93) | 0.208 | 0.677 | 0.070 |
|  | Weighted median | 6 | 0.61 (0.36- 1.04) | 0.067 |  |  |
|  | Inverse variance weighted | 6 | 0.67 (0.41 - 1.10) | 0.111 | 0.139 |  |
|  | Simple mode | 6 | 0.48 (0.20 - 1.16) | 0.162 |  |  |
|  | Weighted mode | 6 | 0.50 (0.22 - 1.12) | 0.152 |  |  |
| RANTES | |  |  |  |  |  |
|  | MR Egger | 10 | 0.52 (0.14 - 1.89) | 0.349 | 0.595 | 0.350 |
|  | Weighted median | 10 | 1.04 (0.52 - 2.08) | 0.912 |  |  |
|  | Inverse variance weighted | 10 | 0.94 (0.56 - 1.59) | 0.828 | 0.589 |  |
|  | Simple mode | 10 | 0.98 (0.31 - 3.08) | 0.969 |  |  |
|  | Weighted mode | 10 | 1.04 (0.34 - 3.14) | 0.952 |  |  |
| MIP-1b | |  |  |  |  |  |
|  | MR Egger | 22 | 2.97 (0.78 - 11.31) | 0.125 | 0.659 | 0.256 |
|  | Weighted median | 22 | 1.36 (0.69- 2.66) | 0.371 |  |  |
|  | Inverse variance weighted | 22 | 1.41 (0.88 - 2.27) | 0.153 | 0.631 |  |
|  | Simple mode | 22 | 1.40 (0.37 - 5.30) | 0.625 |  |  |
|  | Weighted mode | 22 | 1.01 (0.32 - 3.15) | 0.991 |  |  |
| SDF-1a | |  |  |  |  |  |
|  | MR Egger | 9 | 1.90 (0.32 -11.23) | 0.367 | 0.242 | 0.223 |
|  | Weighted median | 9 | 1.04 (0.36- 2.97) | 0.565 |  |  |
|  | Inverse variance weighted | 9 | 0.67 (0.26- 1.69) | 0.394 | 0.175 |  |
|  | Simple mode | 9 | 1.20 (0.27 - 5.26) | 0.868 |  |  |
|  | Weighted mode | 9 | 1.29 (0.31 - 5.41) | 0.834 |  |  |
| IP-10 | |  |  |  |  |  |
|  | MR Egger | 12 | 1.66 (0.40 - 6.89) | 0.500 | 0.059 | 0.779 |
|  | Weighted median | 12 | 1.38 (0.70- 2.73) | 0.349 |  |  |
|  | Inverse variance weighted | 12 | 1.38 (0.75- 2.53) | 0.301 | 0.084 |  |
|  | Simple mode | 12 | 1.40 (0.37 - 5.26) | 0.632 |  |  |
|  | Weighted mode | 12 | 1.38 (0.41 - 4.65) | 0.617 |  |  |
| IL-2ra | |  |  |  |  |  |
|  | MR Egger | 9 | 1.86 (0.77 - 4.51) | 0.210 | 0.648 | 0.171 |
|  | Weighted median | 9 | 1.39 (0.76 - 2.56) | 0.286 |  |  |
|  | Inverse variance weighted | 9 | 1.05 (0.65 - 1.69) | 0.856 | 0.491 |  |
|  | Simple mode | 9 | 1.51 (0.67 - 3.38) | 0.346 |  |  |
|  | Weighted mode | 9 | 1.46 (0.73 - 2.94) | 0.316 |  |  |
| IL-1b | |  |  |  |  |  |
|  | MR Egger | 3 | 0.80 (0.10 -6.41) | 0.868 | 0.466 | 0.982 |
|  | Weighted median | 3 | 0.91 (0.24 - 3.42) | 0.889 |  |  |
|  | Inverse variance weighted | 3 | 0.82 (0.29 - 2.32) | 0.712 | 0.766 |  |
|  | Simple mode | 3 | 0.97 (0.25 - 3.74) | 0.966 |  |  |
|  | Weighted mode | 3 | 0.96 (0.25 - 3.69) | 0.953 |  |  |
| IL-12p70 | |  |  |  |  |  |
|  | MR Egger | 15 | 0.40 (0.04 - 4.58) | 0.477 | 0.249 | 0.725 |
|  | Weighted median | 15 | 0.63 (0.27 - 1.46) | 0.281 |  |  |
|  | Inverse variance weighted | 15 | 0.62 (0.32 - 1.19) | 0.149 | 0.304 |  |
|  | Simple mode | 15 | 0.75 (0.18 - 3.21) | 0.708 |  |  |
|  | Weighted mode | 15 | 0.73 (0.20- 2.69) | 0.645 |  |  |
| MIF | |  |  |  |  |  |
|  | MR Egger | 10 | 3.77 (1.28 - 11.05) | 0.042 | 0.407 | 0.026 |
|  | Weighted median | 10 | 0.80 (0.35 - 1.83) | 0.596 |  |  |
|  | Inverse variance weighted | 10 | 1.04 (0.51- 2.12) | 0.919 | 0.068 |  |
|  | Simple mode | 10 | 0.84 (0.23 - 3.15) | 0.803 |  |  |
|  | Weighted mode | 10 | 0.72 (0.22 - 2.41) | 0.609 |  |  |
| TRAIL | |  |  |  |  |  |
|  | MR Egger | 16 | 1.05 (0.56 - 1.98) | 0.883 | 0.351 | 0.711 |
|  | Weighted median | 16 | 0.83 (0.48 - 1.45) | 0.511 |  |  |
|  | Inverse variance weighted | 16 | 0.96 (0.63 - 1.46) | 0.851 | 0.412 |  |
|  | Simple mode | 16 | 0.71 (0.25 - 2.04) | 0.535 |  |  |
|  | Weighted mode | 16 | 0.82 (0.45 - 1.52) | 0.540 |  |  |
| CCL27 | |  |  |  |  |  |
|  | MR Egger | 12 | 3.92 (1.51 - 10.19) | 0.019 | 0.926 | 0.037 |
|  | Weighted median | 12 | 2.19 (1.10 - 4.34) | 0.025 |  |  |
|  | Inverse variance weighted | 12 | 1.43 (0.88 - 2.33) | 0.147 | 0.513 |  |
|  | Simple mode | 12 | 2.39 (0.70- 8.11) | 0.191 |  |  |
|  | Weighted mode | 12 | 2.43 (0.73 - 8.07) | 0.176 |  |  |
| SCGF-b | |  |  |  |  |  |
|  | MR Egger | 21 | 1.96 (0.96 - 3.98) | 0.079 | 0.448 | 0.486 |
|  | Weighted median | 21 | 1.60 (0.98 - 2.60) | 0.058 |  |  |
|  | Inverse variance weighted | 21 | 1.56 (1.11 - 2.19) | 0.011 | 0.481 |  |
|  | Simple mode | 21 | 2.19 (0.85 - 5.66) | 0.122 |  |  |
|  | Weighted mode | 21 | 1.61 (0.63 - 4.12) | 0.335 |  |  |
| b-NGF | |  |  |  |  |  |
|  | MR Egger | 4 | 0.18 (0.00 - 10.06) | 0.488 | 0.786 | 0.488 |
|  | Weighted median | 4 | 0.87 (0.35 - 2.18) | 0.766 |  |  |
|  | Inverse variance weighted | 4 | 0.97 (0.44 - 2.12) | 0.938 | 0.755 |  |
|  | Simple mode | 4 | 0.81 (0.25 - 2.61) | 0.750 |  |  |
|  | Weighted mode | 4 | 0.84 (0.26- 2.72) | 0.787 |  |  |

Abbreviations: SNP, single nucleotide polymorphism; OR, odds ratio; CI, confidence interval.
