# Supplementary material for: Causal effects of systemic inflammatory proteins on Guillain-Barre Syndrome: insights from genome-wide Mendelian randomization, single-cell RNA sequencing analysis, and network pharmacology
Source: Front Immunol. 2024 Sep 9;15:1456663. doi: 10.3389/fimmu.2024.1456663 (PMC11416972; doi:10.3389/fimmu.2024.1456663)
Supplement: Supplementary file 1 [file DataSheet1.zip › Supplementary materials/Supplementary Table S4.docx]

**Table S4.** The global test and causal estimate results of MR pleiotropy residual sum and outlier (MR-PRESSO).

| Systemic inflammatory proteins | Causal estimate *P*-value | Global test *P*-value | Pleiotropic SNP | FDR-*P*_IVW_ |
| --- | --- | --- | --- | --- |
| CTACK | 0.078 | 0.502 | no outlier | 0.617 |
| β-NGF | 0.165 | 0.539 | no outlier | 0.910 |
| VEGF | 0.262 | 0.933 | no outlier | 0.885 |
| MIF | 0.349 | 0.863 | no outlier | 0.908 |
| TRAIL | 0.281 | 0.725 | no outlier | 0.901 |
| TNF-β | 0.061 | 0.826 | no outlier | 0.857 |
| TNF-α | 0.528 | 0.642 | no outlier | 0.604 |
| SDF-1α | 0.378 | 0.384 | no outlier | 0.809 |
| SCGF-β | 0.238 | 0.474 | no outlier | 0.413 |
| SCF | 0.272 | 0.097 | no outlier | 0.664 |
| IL-16 | 0.541 | 0.325 | no outlier | 0.750 |
| RANTES | 0.627 | 0.308 | no outlier | 0.899 |
| PDGF-bb | 0.052 | 0.713 | no outlier | 0.815 |
| MIP-1β | 0.068 | 0.238 | no outlier | 0.622 |
| MIP-1α | 0.505 | 0.486 | no outlier | 0.871 |
| MIG | 0.511 | 0.075 | no outlier | 0.914 |
| M-CSF | 0.057 | 0.676 | no outlier | 0.519 |
| MCP-3 | 0.335 | 0.343 | no outlier | 0.580 |
| MCP-1 | 0.565 | 0.129 | no outlier | 0.856 |
| IL-12p70 | 0.929 | 0.325 | no outlier | 0.619 |
| IP-10 | 0.183 | 0.464 | no outlier | 0.764 |
| IL-18 | 0.746 | 0.723 | no outlier | 0.870 |
| IL-17 | 0.402 | 0.436 | no outlier | 0.901 |
| IL-13 | 0.484 | 0.778 | no outlier | 0.892 |
| IL-10 | 0.931 | 0.247 | no outlier | 0.867 |
| IL-8 | 0.132 | 0.905 | no outlier | 0.837 |
| IL-6 | 0.353 | 0.580 | no outlier | 0.907 |
| IL-1rα | 0.645 | 0.862 | no outlier | 0.914 |
| IL-1β | 0.034 | 0.956 | no outlier | 0.884 |
| HGF | 0.641 | 0.327 | no outlier | 0.802 |
| IL-9 | 0.453 | 0.755 | no outlier | 0.859 |
| IL-7 | 0.368 | 0.153 | no outlier | 0.428 |
| IL-5 | 0.967 | 0.242 | no outlier | 0.656 |
| IL-4 | 0.508 | 0.642 | no outlier | 0.732 |
| IL-2Rα | 0.252 | 0.476 | no outlier | 0.902 |
| IL-2 | 0.378 | 0.149 | no outlier | 0.914 |
| IFN-γ | 0.776 | 0.068 | no outlier | 0.431 |
| GRO-α | 0.564 | 0.915 | no outlier | 0.868 |
| G-CSF | 0.365 | 0.778 | no outlier | 0.842 |
| b-FGF | 0.347 | 0.267 | no outlier | 0.879 |
| Eotaxin | 0.348 | 0.079 | no outlier | 0.431 |

SNP, single nucleotide polymorphism.
