# Supplementary material for: Causal effects of systemic inflammatory proteins on Guillain-Barre Syndrome: insights from genome-wide Mendelian randomization, single-cell RNA sequencing analysis, and network pharmacology
Source: Front Immunol. 2024 Sep 9;15:1456663. doi: 10.3389/fimmu.2024.1456663 (PMC11416972; doi:10.3389/fimmu.2024.1456663)
Supplement: Supplementary file 1 [file DataSheet1.zip › Supplementary materials/Supplementary Table S5.docx]

**Table S5.** MR estimates of GBS on 41 systemic inflammatory proteins (**Exposure:** GBS).

| Systemic inflammatory proteins (**Outcome**) | nSNP | *P*-value | OR (95%CI) |
| --- | --- | --- | --- |
| CCL27 | 7 | 0.935 | 0.99 (0.86-1.14) |
| β-NGF | 6 | 0.145 | 0.92 (0.81-1.05) |
| VEGF | 6 | 0.136 | 0.95 (0.89-1.04) |
| MIF | 6 | 0.524 | 0.97 (0.88-1.07) |
| TRAIL | 7 | 0.719 | 0.98 (0.92-1.06) |
| TNF-β | 6 | 0.943 | 0.99 (0.87-1.15) |
| TNF-α | 7 | 0.926 | 0.99 (0.88-1.12) |
| SDF-1α | 7 | 0.909 | 1.00 (0.94-1.07) |
| SCGF-β | 6 | 0.294 | 0.93 (0.81-1.09) |
| SCF | 6 | 0.232 | 0.96 (0.89-1.03) |
| IL-16 | 7 | 0.709 | 1.02 (0.93-1.12) |
| RANTES | 7 | 0.489 | 0.96 (0.84-1.07) |
| PDGF-bb | 6 | 0.336 | 1.03 (0.97-1.10) |
| MIP-1β | 7 | 0.337 | 1.03 (0.96-1.10) |
| MIP-1α | 6 | 0.709 | 0.98 (0.89-1.08) |
| CXCL9 | 6 | 0.864 | 0.99 (0.86-1.14) |
| M-CSF | 6 | 0.187 | 0.92 (0.82-1.04) |
| MCP-3 | 7 | 0.332 | 0.91 (0.76-1.10) |
| MCP-1 | 6 | 0.399 | 0.97 (0.91-1.04) |
| IL-12p70 | 6 | 0.539 | 0.98 (0.92-1.04) |
| IP-10 | 6 | 0.521 | 1.04 (0.92-1.17) |
| IL-18 | 6 | 0.292 | 1.05 (0.96-1.16) |
| IL-17 | 7 | 0.314 | 0.97 (0.91-1.03) |
| IL-13 | 6 | 0.143 | 0.93 (0.84-1.02) |
| IL-10 | 6 | 0.152 | 0.95 (0.89-1.02) |
| IL-8 | 6 | 0.116 | 0.93 (0.84-1.02) |
| IL-6 | 6 | 0.717 | 0.99 (0.92-1.06) |
| IL-1rα | 6 | 0.837 | 1.01 (0.92-1.11) |
| IL-1β | 6 | 0.127 | 0.94 (0.87-1.02) |
| HGF | 6 | 0.160 | 0.95 (0.88-1.02) |
| IL-9 | 7 | 0.666 | 1.02 (0.92-1.14) |
| IL-7 | 6 | 0.455 | 0.96 (0.87-1.09) |
| IL-5 | 6 | 0.258 | 0.94 (0.85-1.05) |
| IL-4 | 6 | 0.406 | 0.97 (0.91-1.04) |
| IL-2Rα | 6 | 0.696 | 0.98 (0.87-1.10) |
| IL-2 | 6 | 0.719 | 0.98 (0.89-1.08) |
| IFN-γ | 6 | 0.977 | 0.99 (0.91-1.09) |
| GRO-α | 6 | 0.567 | 0.97 (0.87-1.08) |
| G-CSF | 7 | 0.856 | 0.99 (0.93-1.06) |
| b-FGF | 7 | 0.722 | 0.98 (0.93-1.05) |
| Eotaxin | 6 | 0.664 | 0.99 (0.92-1.08) |
