# Supplementary material for: Causal effects of systemic inflammatory proteins on Guillain-Barre Syndrome: insights from genome-wide Mendelian randomization, single-cell RNA sequencing analysis, and network pharmacology
Source: Front Immunol. 2024 Sep 9;15:1456663. doi: 10.3389/fimmu.2024.1456663 (PMC11416972; doi:10.3389/fimmu.2024.1456663)
Supplement: Supplementary file 1 [file DataSheet1.zip › Supplementary materials/Supplementary Table S6.docx]

**Table S6.** Characteristics of instrumental variables used for IFN-γ, IL-7, SCGF-β, and Eotaxin in this study.

| Inflammatory  proteins | SNP | Chr | Position | Effect allele | Beta | SE | *P*-value | |
| --- | --- | --- | --- | --- | --- | --- | --- | --- |
| IFN-γ | rs113399544 | 7 | 149364875 | A | -0.0849 | 0.0183 | 3.323E-06 | |
| IFN-γ | rs113600793 | 17 | 45620741 | A | 0.1871 | 0.0371 | 4.426E-07 | |
| IFN-γ | rs115729819 | 4 | 169704667 | A | 0.2511 | 0.0514 | 1.045E-06 | |
| IFN-γ | rs117046255 | 7 | 106484822 | T | -0.0968 | 0.0207 | 2.787E-06 | |
| IFN-γ | rs11843756 | 13 | 49254892 | T | 0.1812 | 0.0391 | 3.622E-06 | |
| IFN-γ | rs12420286 | 11 | 103777894 | T | 0.2357 | 0.05 | 2.452E-06 | |
| IFN-γ | rs147378920 | 1 | 22701995 | A | -0.384 | 0.0751 | 3.195E-07 | |
| IFN-γ | rs1867282 | 9 | 102172147 | T | 0.0781 | 0.0166 | 2.478E-06 | |
| IFN-γ | rs2073438 | 17 | 6900076 | A | 0.092 | 0.0188 | 9.551E-07 | |
| IFN-γ | rs7088799 | 10 | 65016174 | T | -0.0805 | 0.0166 | 1.274E-06 | |
| IFN-γ | rs73479333 | 6 | 82980783 | C | -0.1123 | 0.024 | 2.816E-06 | |
| IFN-γ | rs74148555 | 10 | 92079842 | T | -0.3771 | 0.077 | 9.858E-07 | |
| IL-7 | rs117509142 | 8 | 87134083 | T | -0.3213 | 0.0684 | 2.599E-06 |  |
| IL-7 | rs11757972 | 6 | 43858592 | T | 0.121 | 0.0257 | 2.529E-06 |  |
| IL-7 | rs1374279 | 2 | 168652203 | A | 0.1625 | 0.0347 | 2.792E-06 |  |
| IL-7 | rs142397827 | 5 | 17484169 | A | 0.4592 | 0.0994 | 3.822E-06 |  |
| IL-7 | rs17091524 | 14 | 56948759 | T | 0.5092 | 0.1015 | 5.244E-07 |  |
| IL-7 | rs2006957 | 6 | 43913243 | T | 0.2557 | 0.0262 | 1.434E-22 |  |
| IL-7 | rs218238 | 4 | 55395024 | A | 0.1319 | 0.0284 | 3.277E-06 |  |
| IL-7 | rs28793375 | 8 | 41415618 | T | 0.1644 | 0.036 | 4.866E-06 |  |
| IL-7 | rs62006410 | 14 | 103007935 | T | -0.1492 | 0.0302 | 7.588E-07 |  |
| IL-7 | rs7155170 | 14 | 30338797 | A | -0.1236 | 0.027 | 4.787E-06 |  |
| IL-7 | rs77318030 | 19 | 55055897 | T | -0.2966 | 0.0631 | 2.639E-06 |  |
| IL-7 | rs77981494 | 16 | 17544866 | T | -0.5201 | 0.1055 | 8.225E-07 |  |
| SCGF-β | rs11111869 | 12 | 104402485 | A | 0.1621 | 0.0311 | 1.861E-07 |  |
| SCGF-β | rs112346514 | 19 | 12407988 | T | -0.3261 | 0.0703 | 3.543E-06 |  |
| SCGF-β | rs1149926 | 10 | 9190949 | T | -0.3458 | 0.0749 | 3.917E-06 | |
| SCGF-β | rs118003677 | 12 | 99755839 | T | -0.3654 | 0.0786 | 3.348E-06 | |
| SCGF-β | rs12118918 | 1 | 169270660 | A | -0.1631 | 0.035 | 3.208E-06 | |
| SCGF-β | rs12480722 | 20 | 20228904 | T | 0.1654 | 0.0353 | 2.812E-06 | |
| SCGF-β | rs13287050 | 9 | 78810117 | A | -0.121 | 0.0263 | 4.118E-06 | |
| SCGF-β | rs13866 | 19 | 51228746 | T | -0.1647 | 0.028 | 3.773E-09 | |
| SCGF-β | rs139413256 | 7 | 145879644 | A | -0.5174 | 0.1076 | 1.532E-06 | |
| SCGF-β | rs143829871 | 3 | 47597245 | T | -0.1866 | 0.0399 | 2.852E-06 | |
| SCGF-β | rs144724875 | 19 | 51195936 | T | 0.5381 | 0.0829 | 8.645E-11 | |
| SCGF-β | rs149009264 | 10 | 45130285 | A | 0.4551 | 0.0985 | 3.793E-06 | |
| SCGF-β | rs150733161 | 13 | 67799297 | T | -0.5255 | 0.112 | 2.687E-06 | |
| SCGF-β | rs151194174 | 7 | 20995778 | A | 0.4536 | 0.0941 | 1.454E-06 | |
| SCGF-β | rs264157 | 18 | 10941746 | A | 0.1079 | 0.0233 | 3.685E-06 | |
| SCGF-β | rs34911860 | 1 | 80350715 | A | -0.3674 | 0.0787 | 3.002E-06 | |
| SCGF-β | rs3817303 | 12 | 104152856 | T | 0.1362 | 0.0294 | 3.602E-06 | |
| SCGF-β | rs4737731 | 8 | 66333562 | T | 0.1146 | 0.0251 | 4.871E-06 | |
| SCGF-β | rs4976691 | 5 | 176827815 | C | -0.1484 | 0.0253 | 4.438E-09 | |
| SCGF-β | rs77954165 | 9 | 6739753 | T | 0.2631 | 0.0562 | 2.867E-06 | |
| SCGF-β | rs78217154 | 8 | 101554072 | T | 0.3942 | 0.0861 | 4.722E-06 | |
| Eotaxin | rs11087905 | 21 | 25505329 | A | 0.0954 | 0.0188 | 4.07E-07 | |
| Eotaxin | rs112347425 | 3 | 46460888 | T | 0.1595 | 0.0276 | 7.771E-09 | |
| Eotaxin | rs11920996 | 3 | 42542761 | T | 0.2979 | 0.0377 | 2.919E-15 | |
| Eotaxin | rs1677588 | 1 | 91337172 | T | 0.1181 | 0.025 | 2.223E-06 | |
| Eotaxin | rs2024050 | 7 | 75460393 | A | 0.164 | 0.0302 | 5.467E-08 | |
| Eotaxin | rs2027855 | 22 | 47837513 | T | 0.0743 | 0.0162 | 4.272E-06 | |
| Eotaxin | rs2040143 | 21 | 18041107 | A | -0.0858 | 0.0178 | 1.333E-06 | |
| Eotaxin | rs2229593 | 3 | 42906216 | T | 0.3647 | 0.0406 | 2.838E-19 | |
| Eotaxin | rs2249581 | 1 | 159144581 | T | -0.0899 | 0.018 | 5.912E-07 | |
| Eotaxin | rs5754733 | 22 | 34269594 | A | -0.105 | 0.0213 | 8.196E-07 | |
| Eotaxin | rs57723662 | 17 | 32535816 | C | -0.0982 | 0.0213 | 3.878E-06 | |
| Eotaxin | rs60075014 | 5 | 31846437 | T | -0.1688 | 0.0356 | 2.078E-06 | |
| Eotaxin | rs7231030 | 18 | 56906961 | A | 0.0903 | 0.0193 | 2.709E-06 | |
| Eotaxin | rs73072941 | 3 | 43307535 | A | -0.1281 | 0.0263 | 1.113E-06 | |
| Eotaxin | rs745331 | 15 | 27929147 | A | -0.0821 | 0.0176 | 3.036E-06 | |
| Eotaxin | rs75426604 | 14 | 35857714 | A | -0.1371 | 0.0291 | 2.397E-06 | |
| Eotaxin | rs9317045 | 13 | 59630038 | A | 0.1172 | 0.0236 | 6.954E-07 | |

Abbreviations: Chr, chromosome; SE, standard error; SNP, single nucleotide polymorphism.
