# Supplementary material for: Causal effects of systemic inflammatory proteins on Guillain-Barre Syndrome: insights from genome-wide Mendelian randomization, single-cell RNA sequencing analysis, and network pharmacology
Source: Front Immunol. 2024 Sep 9;15:1456663. doi: 10.3389/fimmu.2024.1456663 (PMC11416972; doi:10.3389/fimmu.2024.1456663)
Supplement: Supplementary file 1 [file DataSheet1.zip › Supplementary materials/Supplementary Table S7.docx]

**Table S7.** Free binding energy and RMSD of the key active components of anti-inflammatory or neuroprotective small molecular compounds and target protein (IL-7).

| **Component** | **Target** | **Free binding energy** (kcal/mol) | **RMSD** |
| --- | --- | --- | --- |
| Stigmasterol | IL-7 | -6.4 | 0.656 |
| Saffronin | IL-7 | -5.8 | 0.176 |
| Quercetin | IL-7 | -5.8 | 1.400 |
| Kaempferol | IL-7 | -5.4 | 1.949 |
| Naringenin | IL-7 | -5.1 | 1.897 |

RMSD, Root Mean Square Deviation.
